# Multimodal Transformer for Sample-Aware Prediction of Metal–Organic Framework Properties

**DOI:** 10.1021/acscentsci.6c01049

**Published:** 2026-09-01

**Authors:** Seunghee Han, Jaewoong Lee, Jihan Kim

**Affiliations:** † Department of Chemical and Biomolecular Engineering, 34968 Korea Advanced Institute of Science and Technology, Daejeon 34141, Republic of Korea; ‡ Department of Materials Science and Engineering, 34968 Korea Advanced Institute of Science and Technology, Daejeon 34141, Republic of Korea

## Abstract

Metal–organic frameworks (MOFs) are a major target of machine-learning-based property prediction, yet most models assume that a single framework representation maps to a single property value. This assumption becomes problematic for experimental MOFs, where samples reported as the same framework can exhibit different properties because of differences in crystallinity, phase purity, defects, and other sample-dependent factors. Here we introduce Experimental X-ray Diffraction Integrated Transformer (EXIT), a multimodal transformer for sample-aware prediction of MOF properties that combines MOFid with X-ray diffraction (XRD). In EXIT, MOFid encodes MOF identity, whereas XRD provides complementary information about the experimentally realized sample state. EXIT is pretrained on one million hypothetical MOFs with simulated XRD to learn transferable representations. The pretrained model is first evaluated through fine-tuning on downstream tasks using simulated XRD, where it achieves improved performance relative to existing approaches, and is subsequently fine-tuned on literature-derived experimental XRD–property data sets for surface area and pore volume prediction. Incorporating experimental XRD improves predictive performance relative to models without experimental XRD, and attention analysis and sample-level case studies further show that EXIT assigns different predictions to samples sharing the same MOF identity when their XRD patterns differ. These results establish a practical step from framework-aware to sample-aware MOF property prediction and highlight the value of incorporating experimental characterization into porous materials informatics.

## Graphical abstract

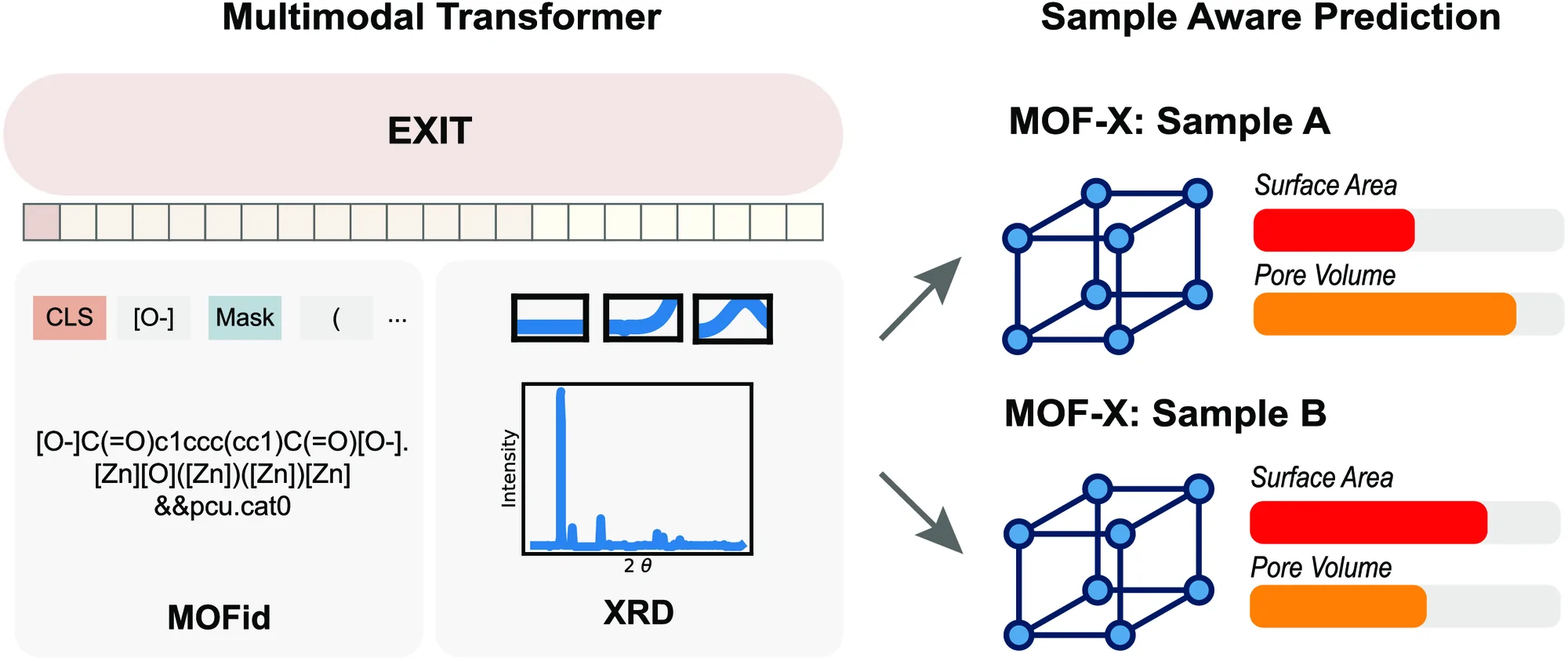

## Introduction

Machine learning has become an important tool for predicting material properties across a wide range of systems,^1^ including zeolites,^2,3^ metal–organic frameworks (MOFs),^4,5^ polymers,^6,7^ and inorganic solids.^8^ In many of these settings, models learn a mapping from a material representation to a scalar property value and have shown strong performance when trained on large, curated data sets.^9,10^ This formulation is especially natural for simulation-ready databases, where each material is represented by a well-defined structure and paired with properties computed under idealized conditions.^11–13^ However, the same formulation is less straightforward for experimental materials data, where the relationship between a nominal material identity and a measured property is often not strictly one-to-one.^14,15^

A central difficulty is that experimental measurements are performed not on an abstract material label, but on a realized sample. As a result, the same nominal material can exhibit different reported properties across studies because of differences in synthesis conditions, activation procedures, crystallinity, defect concentrations, and other sample-dependent factors.^16–18^ In this sense, part of the discrepancy between machine-learning predictions and experimental measurements is not simply noise, but a representation problem: when the model input encodes only composition, topology, or an idealized structure, sample-level variation may be forced into the residual error rather than represented explicitly.^19,20^ This issue is particularly visible in MOFs, where even widely studied frameworks such as MOF-5, HKUST-1, and UiO-66 are known to show substantial spreads in experimentally reported properties.^18,21–23^

To this end, MOFs provide a useful setting in which to study this problem because their structural diversity has made them a major target of ML-based property prediction, while at the same time, their experimentally measured properties often depend strongly on sample history and preparation.^24,25^ Existing ML models for MOFs typically rely on descriptors or encoded features derived from ideal crystal structures.^10,26,27^ Yet such representations are fundamentally framework-level, as they encode the nominal identity of the MOF but not necessarily the state of the experimentally realized sample. For experimental MOF prediction, this creates a gap between the information available to the model and the factors that determine the reported measurement.

One practical way to reduce this mismatch is to augment framework- or identifier-based representations with an experimental descriptor that reflects sample state. Powder X-ray diffraction (XRD) is an attractive candidate for this purpose because it is routinely reported in MOF synthesis studies and is sensitive to crystallinity, phase composition, symmetry, structural distortion, and other characteristics of the realized sample that are not fully captured by nominal framework identity alone.^28^ XRD is also appealing from a modeling perspective because it can be incorporated in both simulated and experimental settings. In hypothetical MOFs, diffraction patterns can be readily generated from crystal structures, enabling large-scale multimodal pretraining with paired MOF representations and XRD data. Prior studies have shown that simulated XRD patterns can support prediction of properties in inorganic crystals and porous materials.^29,30^ For experimental MOFs, XRD is routinely reported as a standard characterization following synthesis, making it readily accessible for literature-based data collection.

Here we introduce EXIT (Experimental X-ray Diffraction Integrated Transformer), a multimodal framework for MOF property prediction that combines MOFid^31^ with XRD information. EXIT is designed to integrate MOF-level representations with diffraction data that reflect sample-dependent characteristics. We first pretrain EXIT using large-scale MOFid–simulated-XRD pairs, evaluate the transferability of the learned representations through simulated-XRD fine-tuning for thermal decomposition temperature and CH_4_ uptake, and then fine-tune the model using literature-derived experimental XRD–property data sets for surface area and pore volume prediction. Our goal here is not to claim that diffraction fully resolves the complexity of experimental variability, but rather to test whether it provides useful sample-level information beyond MOF identity alone. By treating MOF property prediction as a sample-aware problem, this work explores a practical strategy to narrow the gap between idealized MOF representations and experimentally measured behavior.

## Results

### EXIT: Multimodal Architecture and Pretraining

The overall workflow of EXIT is illustrated in Figure 1. EXIT is a multimodal Transformer model designed to integrate two complementary inputs: MOFid and an XRD representation. MOFid encodes the idealized chemical identity of a framework in a language-like format, including the metal node, organic linker, topology, and catenation,^31^ whereas XRD provides complementary information on the experimentally realized crystalline state, such as phase identity, symmetry, crystallinity, crystallite domain size, strain, and preferred orientation. In the architecture used in this study, MOFid is tokenized as a sequence input, and XRD is encoded by a one-dimensional convolutional neural network before multimodal fusion.

**Figure 1:**
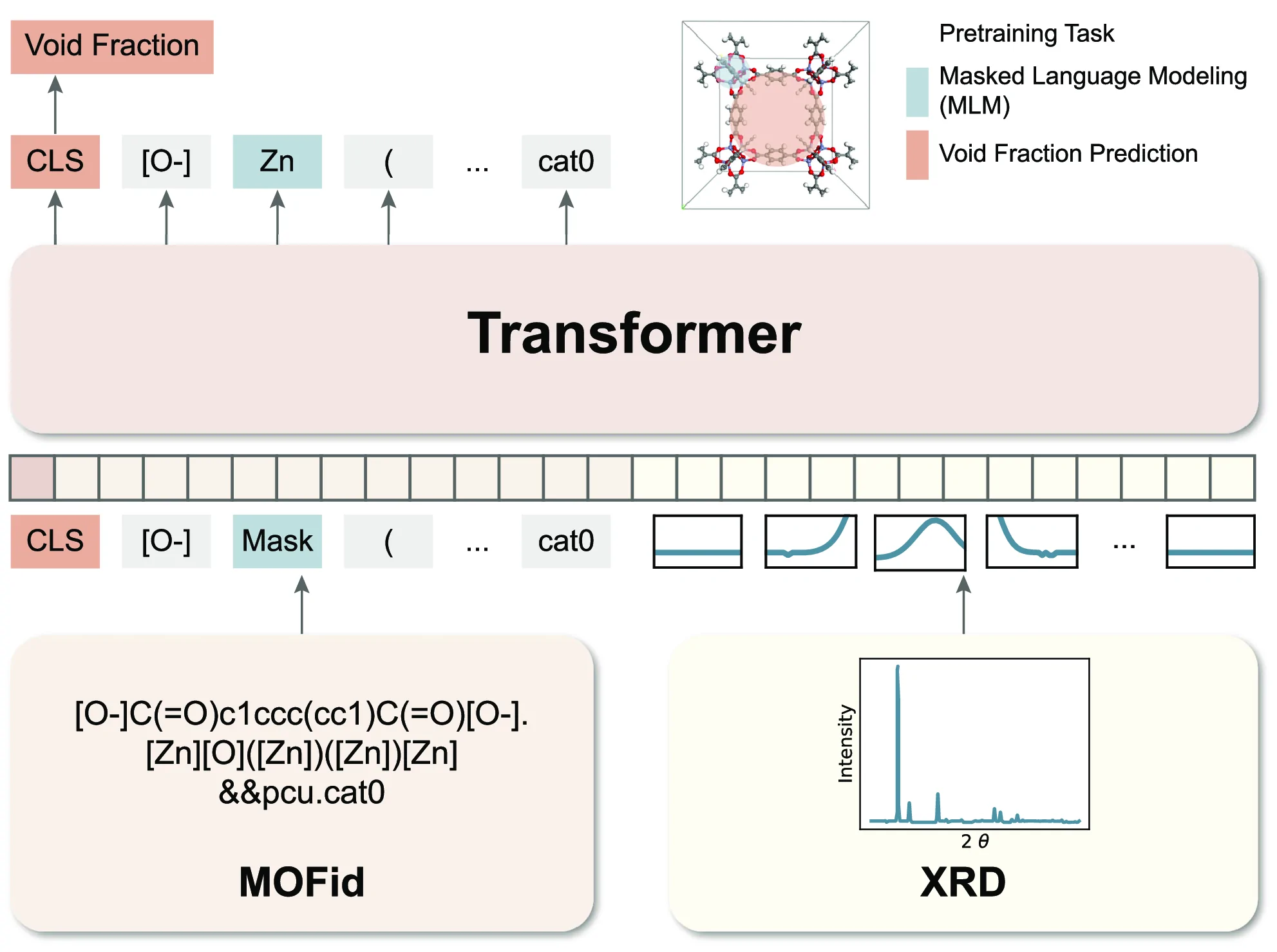
Overview of the EXIT framework. MOF structures are represented using MOFid, while X-ray diffraction (XRD) patterns are incorporated as an additional modality reflecting experimentally accessible structural information. The MOFid sequence and discretized XRD signals are jointly embedded and processed by a Transformer encoder. During pretraining, the model is trained on MOFid–simulated-XRD pairs using masked language modeling (MLM) on MOFid tokens and a regression task for predicting void fraction from the [CLS] representation. This multimodal pretraining enables the model to learn representations that integrate framework identity with diffraction-derived features.

EXIT was first pretrained to learn universal representations of MOFs. Because large-scale paired experimental MOFid–XRD data sets are not available, we constructed a pretraining data set by combining hypothetical MOFs generated with PORMAKE^11^ and MOF structures collected from the hMOF,^12^ CoRE MOF,^32^ and QMOF databases.^33^ Simulated XRD patterns for all structures were then computed using pymatgen.^34^ The pretraining tasks consisted of masked language modeling (MLM) on MOFid and void-fraction prediction using the [CLS] token. In MLM, randomly selected MOFid tokens are masked and predicted from context. The [CLS] token is a special learnable token whose final hidden representation summarizes the combined MOFid–XRD input. Together, these tasks allowed the model to learn both framework-level chemical representations and global structure-related features from the XRD data. The hypothetical data set was split into training and test sets at a ratio of 0.95:0.05, and the pretraining performance is summarized in Table S1.

### Effect of Pretraining on Downstream Tasks with Simulated XRD

To assess the effect of pretraining and benchmark EXIT against prior models, we performed downstream prediction tasks using simulated XRD for thermal decomposition temperature (T_D_)^35^ and CH_4_ uptake at 298 K and 2.5 bar.^12^ The T_D_ data set contained 3,022 experimentally measured values, whereas the CH_4_ data set contained 120,653 simulated values. For each task, we evaluated representative baselines from previous studies, including descriptor-based models, MOFormer,^27^ and MOFTransformer.^10^ Pretrained EXIT achieved the lowest MAE for T_D_ and performance comparable to MOFTransformer for CH_4_ uptake (Table 1). Compared with EXIT trained from scratch, pretraining substantially improved accuracy: the mean absolute error (MAE) decreased from 54.99 to 44.58 K for T_D_ and from 0.30 mol kg^–1^ to 0.17 mol kg^–1^ for CH_4_ uptake. For T_D_, however, the improvement over descriptor + RF was small, suggesting that additional model complexity provides limited benefit for this relatively small and experimentally measured data set. In contrast, the benefit of pretraining was more evident for the substantially larger simulated CH_4_ data set. These results suggest that the advantage of model capacity and pretraining depends on the size and characteristics of the downstream data set.

**Table 1:** Effect of Multimodal Pretraining on Property Prediction Using Simulated XRD^a^

Mean Absolute Error (MAE)	Thermal Decomposition Temperature (T_D_)	CH_4_ Adsorption (298 K, 2.5 bar)
Descriptor + Random Forest (RF)	45.03	0.21
MOFormer	50.14	0.33
MOFTransformer	48.43	**0.17**
EXIT (scratch)	54.99	0.30
EXIT (pretrained)	**44.58 (↓)**	**0.17(↓)**

a Mean absolute error (MAE) is reported in K for thermal decomposition temperature (T_D_) and in mol kg^–1^ for CH_4_ adsorption at 298 K and 2.5 bar. The table compares representative baseline models with EXIT trained from scratch and after pre-training. Pre-training improved EXIT performance on both tasks.

We further conducted an ablation study to examine how different pretraining tasks affected downstream performance (Table S2). Masked language modeling contributed more strongly to T_D_ prediction, whereas void-fraction prediction was more beneficial for CH_4_ uptake prediction. Nevertheless, combining MOFid masked language modeling and void-fraction pretraining outperformed either task alone. Taken together, these results show that multimodal pretraining provides an effective initialization for downstream tasks and allows EXIT to learn transferable structure–property relationships before exposure to experimental data.

### Construction of the Experimental Data Set

Having validated EXIT on downstream tasks with simulated XRD, we next constructed an experimental data set to fine-tune the model for prediction of experimentally measured properties. The overall workflow of experimental data set construction is shown in Figure 2.

**Figure 2:**
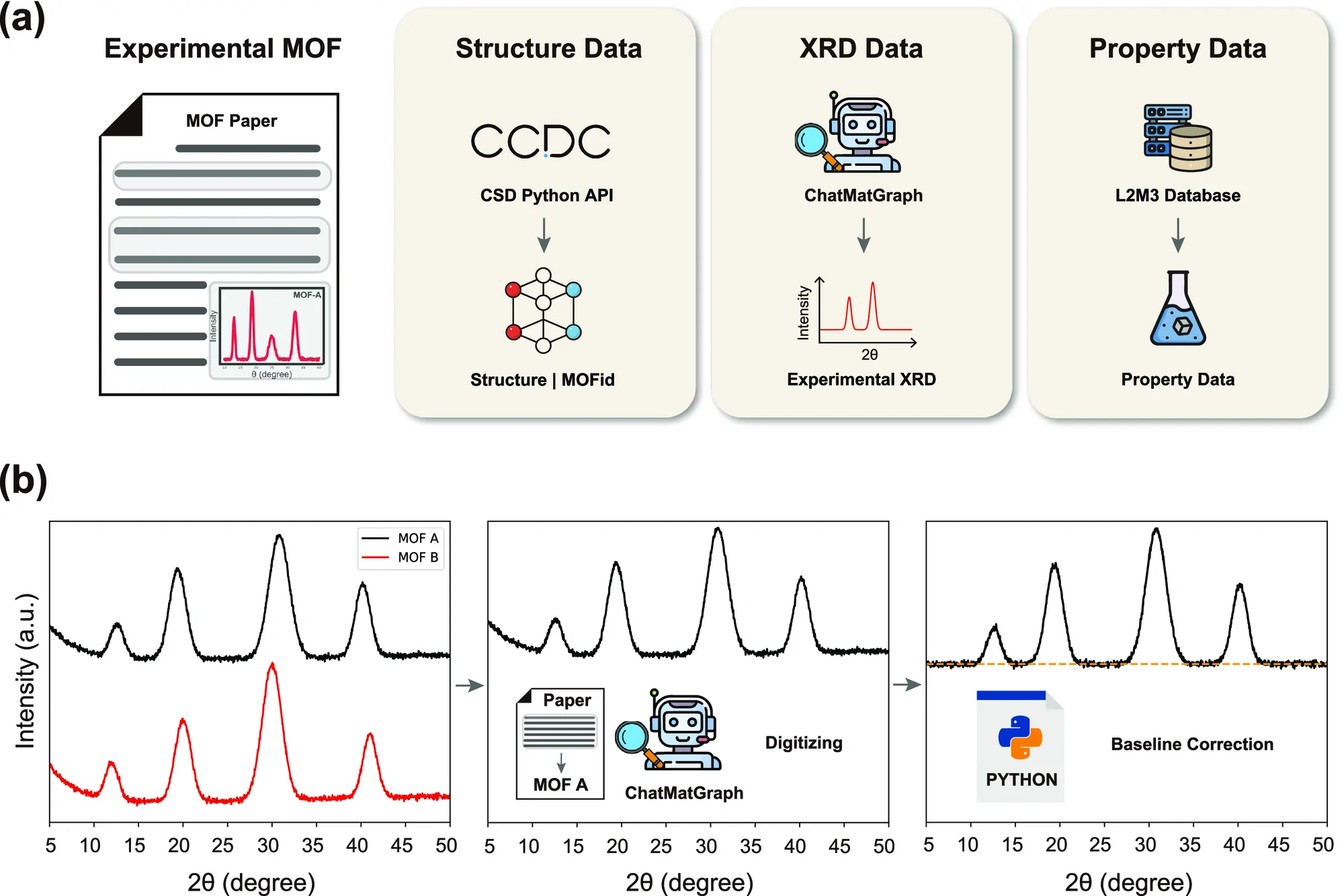
Workflow for construction of the experimental MOF data set. (a) Experimental XRD, structure, and property data were collected from the literature using MatGD with LLM, CCDC records, and the L2M3 database. (b) XRD patterns were extracted by panel separation and digitization, followed by baseline correction.

Experimental XRD patterns were obtained using MatGD,^36^ with the multimodal large language model GPT-5.4. We started from 69,183 MOF-related papers whose DOIs were listed in the large language model–based MOF miner (L2M3) literature database reported in our previous work.^24^ For each paper, figure images were downloaded from ACS and Elsevier journals using the corresponding DOI, and GPT-5.4 was used to identify figures containing XRD patterns. When a single figure contained multiple XRD panels, MatGD separated them into individual images, which were then digitized into numerical diffraction patterns. The extracted patterns were subsequently processed by baseline correction, rule-based postprocessing, and min–max normalization to the range of 0 to 1. Cases in which XRD extraction failed were discarded. MOF identities were assigned primarily from graph legends using an LLM, with caption information incorporated by the LLM as supplementary context. Further details of the MatGD-LLM pipeline are provided in the Methods section.

In parallel, experimental MOFs were mapped to framework identities by checking DOI–name–refcode pairs, retrieving CIF structures through CCDC references when available, refining the structures, removing free solvent, and filtering invalid entries using MOFChecker. Additional manual refinement was applied, where possible, to correct invalid CIF files and to resolve naming ambiguities for representative MOFs such as MOF-5 and HKUST-1. MOFid was then extracted from the curated CIF structures.

Property data were collected for surface area (SA) and pore volume (PV) from L2M3^24^. For surface area, only Brunauer–Emmett–Teller (BET) values reported in m^2^ g^–1^ were retained. For pore volume, only total pore volume values reported in cm^3^ g^–1^ were retained. Records associated with nonstandard units, value ranges, lists, or ambiguous duplicate entries were removed during curation. Despite the large initial literature corpus, the final number of usable samples was limited by the requirement that each sample include a matched MOFid, an experimental XRD pattern, and a curated property value. Many candidate entries lacked at least one of these components, for example an associated CIF structure, an extractable XRD figure, or a usable reported property value. In addition, some XRD-containing figures could not be successfully parsed by MatGD, especially when multiple diffraction panels overlapped within a single figure. The final curated data set comprised 311 surface area records across 84 MOFs and 181 pore volume records across 49 MOFs, each paired with MOFid and experimental XRD. The relatively small size and literature-derived nature of the data set introduce heterogeneity in synthesis and activation procedures, sample handling, and property-measurement protocols across studies. In addition, recovering XRD patterns from published figures may introduce uncertainty associated with image resolution, overlapping traces, baseline correction, and resampling. Although all extracted patterns were manually inspected and ambiguous cases were removed, digitization uncertainty cannot be eliminated entirely.

### Experimental XRD and MOF Property Prediction

Because most previous MOF property predictors rely only on idealized framework information, they cannot distinguish samples that share the same nominal MOF identity but differ in experimentally realized state. To address this limitation, we incorporated experimentally measured XRD into EXIT to improve prediction of literature-derived experimental properties. The curated surface area and pore volume data sets were split into training, validation, and test sets at a ratio of 0.8:0.1:0.1, and the pretrained EXIT model was fine-tuned on each task. The validation fold was used for checkpoint selection during fine-tuning. For comparison, corresponding models were also trained without experimental XRD, where the experimental pattern for each sample was replaced by the simulated XRD pattern generated from its corresponding idealized framework. Thus, both models retained MOFid and XRD as input modalities, and the comparison was designed to isolate the contribution of sample-specific information contained in experimental XRD. For surface area prediction, replacing framework-derived simulated XRD with sample-specific experimental XRD improved the test performance from R^2^ = 0.30 and MAE = 405 m^2^ g^–1^ to R^2^ = 0.53 and MAE = 334 m^2^ g^–1^. For pore volume prediction, the same replacement improved test performance from R^2^ = 0.12 and MAE = 0.26 cm^3^ g^–1^ to R^2^ = 0.59 and MAE = 0.22 cm^3^ g^–1^ (Figure 3). Given the small and heterogeneous nature of the experimental data sets, these performance values should be interpreted as evidence that experimental XRD contains predictive information beyond MOF identity within the present literature-derived data set, rather than as a definitive estimate of generalization performance across all MOFs, literature sources, and experimental conditions.

**Figure 3:**
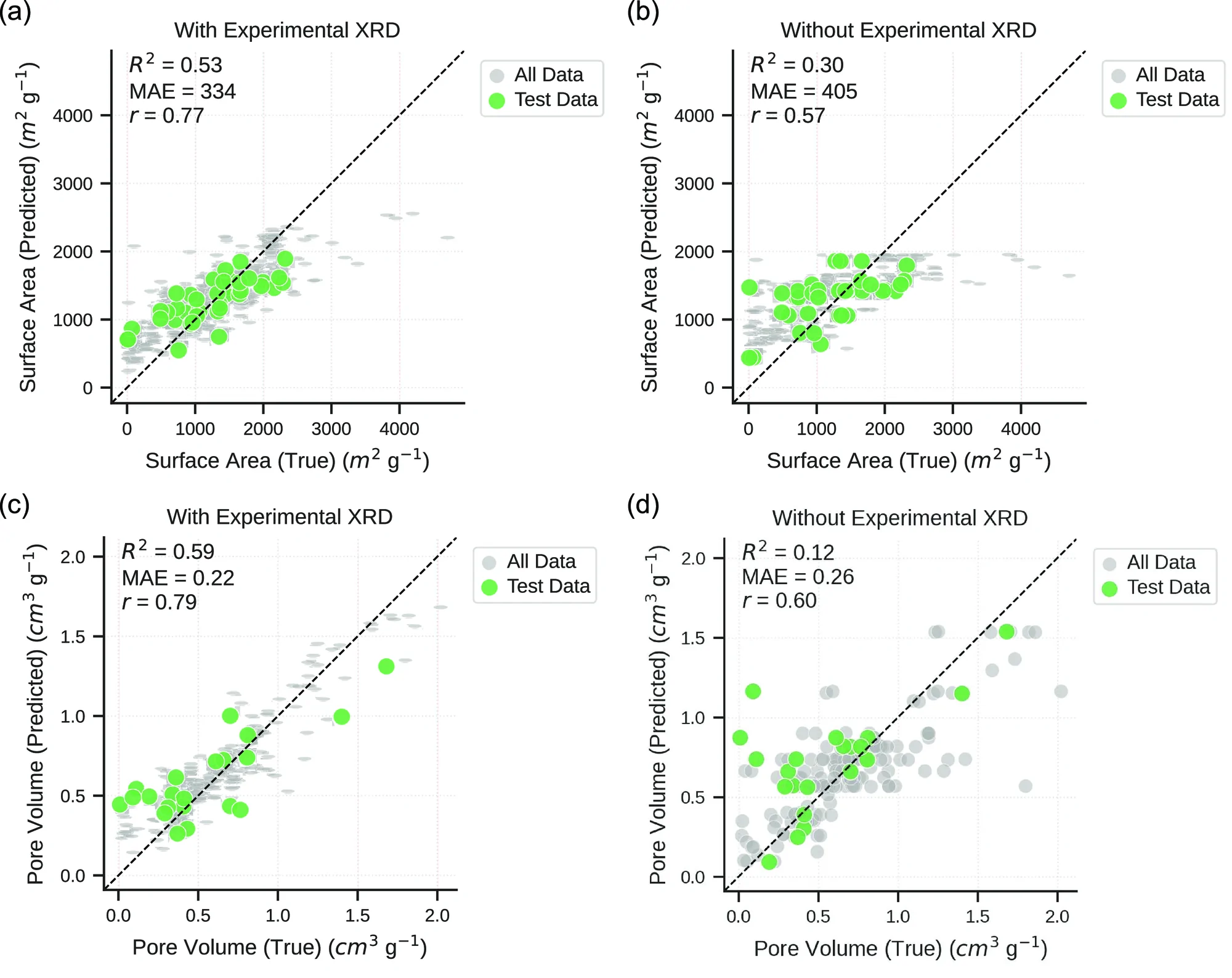
Prediction results of EXIT for experimental surface area (SA) and pore volume (PV). Scatter plots compare predicted and true values for models trained (a, c) with and (b, d) without experimental XRD. In the models without experimental XRD, each sample-specific experimental pattern was replaced by the simulated XRD pattern generated from the corresponding idealized framework. Panels (a) and (b) show SA prediction, and panels (c) and (d) show PV prediction. Gray points represent all data, and green points indicate the test set. The dashed line represents the identity line. R^2^, mean absolute error (MAE), and Pearson correlation coefficient (*r*) are reported for the test set. MAE values are given in m^2^ g^–1^ for SA and cm^3^ g^–1^ for PV.

We further evaluated model robustness using 9-fold cross-validation. The full data set was divided into ten folds, with one fold fixed as the test set and the remaining nine used for cross-validation. Across the nine folds, models using experimental XRD achieved mean R^2^ values of 0.45 ± 0.11 for surface area and 0.47 ± 0.08 for pore volume, compared with 0.20 ± 0.10 and 0.12 ± 0.10, respectively, for the corresponding models using framework-derived simulated XRD. Consistent with the test-set results, models incorporating experimental XRD performed better on average than the corresponding comparison models for both surface area and pore volume (Tables S3 and S4).

The predictive improvement was also evident relative to a simple zero-order estimate based on the data set mean. Assigning the data set mean to every sample gave MAE values of 555 m^2^ g^–1^ for surface area and 0.30 cm^3^ g^–1^ for pore volume. This improvement is particularly meaningful because variations in surface area and pore volume can directly affect adsorption-related performance. This is also practically relevant because powder XRD is routinely collected during MOF synthesis and characterization, whereas direct measurement of SA and PV typically requires additional gas sorption experiments. As a result, XRD-informed prediction may help prioritize which experimentally synthesized samples warrant further characterization or application-specific testing. For example, H_2_ uptake is often correlated with BET surface area, consistent with the empirical Chahine’s rule, such that even samples with the same MOF may exhibit different gas adsorption or separation behavior.^37^

### Analysis of Experimental XRD Information

To better understand how experimental XRD enables sample-specific prediction, we examined two representative MOF-808 samples with different measured pore volumes (Figure 4). When sample-specific experimental XRD was provided, EXIT produced distinct pore volume predictions for the two samples. In contrast, when the experimental patterns were replaced by the simulated XRD pattern generated from the corresponding idealized MOF-808 framework, the samples received the same predicted value of approximately 0.87 cm^3^ g^–1^. Because the two samples share identical MOFid and framework-derived simulated-XRD inputs in this comparison, the model cannot distinguish their sample-specific states without experimental XRD.

**Figure 4:**
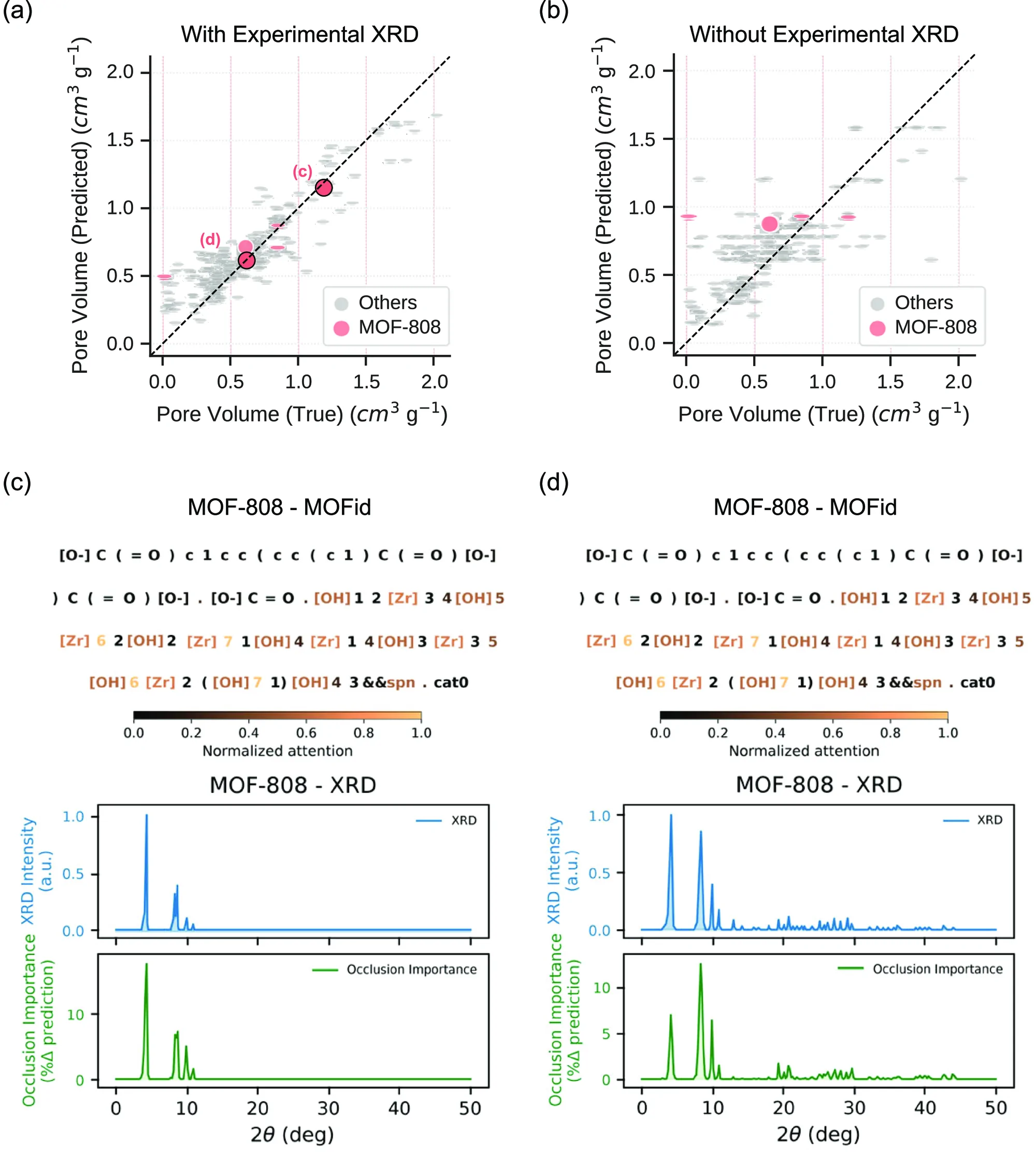
Case study of MOF-808 pore volume (PV) prediction and patch-wise XRD occlusion sensitivity using EXIT. Panels (a) and (b) compare predicted and true PV values for models trained with and without experimental XRD, respectively, with MOF-808 samples highlighted in red; the dashed line in each panel indicates the identity line. Panels (c) and (d) show the MOFid attention maps, experimental XRD patterns, and patch-wise occlusion sensitivity profiles for the circled MOF-808 samples in panel (a). In the MOFid plots, lighter colors indicate higher normalized attention weights. Occlusion sensitivity represents the absolute percentage change in predicted PV when each XRD patch is individually set to the normalized baseline while the MOFid input is held fixed.

The MOFid attention maps remained similar for the two samples, consistent with their shared framework identity, whereas their experimental XRD patterns exhibited distinct sample-dependent features. To identify which diffraction features directly influenced the predictions, we performed a patch-wise occlusion sensitivity analysis while holding the MOFid input fixed. Specifically, each XRD patch was individually set to zero, corresponding to the normalized baseline, and the perturbed input was passed through the trained model. Occlusion sensitivity was quantified as the absolute percentage change in predicted pore volume relative to the unperturbed prediction. For the samples shown in Figures 4c and 4d, the largest changes in predicted pore volume were 17.52% at 2θ ≈ 4.3° and 12.45% at 2θ ≈ 8.3°, respectively, both coinciding with prominent low-angle diffraction peaks. These results indicate that EXIT uses sample-specific diffraction features to distinguish different samples associated with the same nominal framework.

Beyond this sample-level analysis, we next examined whether the pretrained attention representations also capture a broader distinction between simulated and experimental XRD. For the simulated data set, 1,000 samples were randomly selected from the pretraining test set, whereas the experimental data set consisted of the samples used for the surface area (SA) and pore volume (PV) tasks. Attention representations extracted from the pretrained EXIT model were projected using t-SNE, and simulated and experimental samples were clearly separated in the resulting embedding space (Figure 5a).

**Figure 5:**
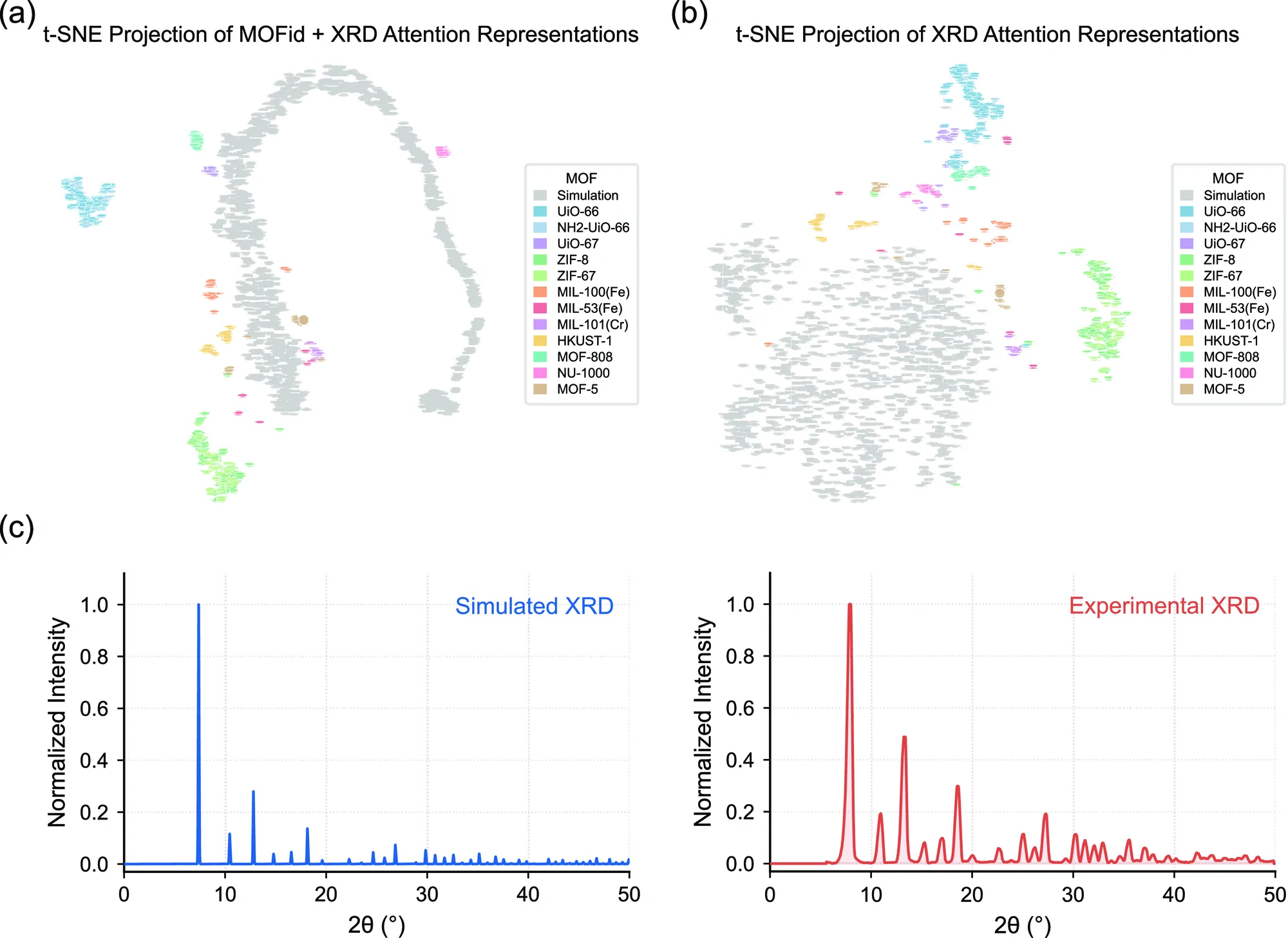
t-SNE visualization of attention representations from the pretrained EXIT model. (a) and (b) show two-dimensional projections of attention representations obtained from MOFid + XRD and XRD-only inputs, respectively. Colored points denote experimental samples from the SA and PV data sets, while gray points denote 1,000 random samples from the pretraining test set with simulated XRD. (c) compares simulated and experimental XRD patterns for ZIF-8, highlighting differences between idealized and experimentally measured diffraction signals.

Because the combined attention representation may still be influenced by both modalities, we additionally performed t-SNE using only the XRD attention representations. In this case, the separation between simulated and experimental samples became even more distinct, with clearer clustering observed for the two groups (Figure 5b). These results indirectly indicate that experimental XRD differs systematically from simulated XRD. Consistent with this interpretation, a representative ZIF-8 example shows that experimental XRD can differ substantially from the corresponding simulated XRD pattern, likely due to factors such as crystallite size, defects, guest or solvent inclusion, microstrain, and activation state (Figure 5c).

### MOF-Dependent Interpretability and Limitations of EXIT

Because sample-to-sample variation in experimentally reported MOF properties may partly originate from differences in synthesis conditions, we next examined whether the available synthesis metadata could account for the observed variation. We analyzed ZIF-8 as a representative case study because it has been synthesized using multiple Zn precursors and solvents, including Zn nitrate and Zn acetate as precursors and H_2_O, methanol (MeOH), and DMF + MeOH as solvents. Synthesis conditions were first extracted using L2M3, which recovered both solvent and precursor information for 9 of the 25 ZIF-8 records. The source articles for the remaining records were then manually reviewed using their DOIs, yielding both annotations for an additional 13 records. Neither field could be established for the remaining 3 records. Samples with unknown annotations or belonging to categories represented by a single sample were excluded from the grouped analyses. The complete solvent and precursor breakdown for all 25 ZIF-8 records is provided in Table S7. Consequently, the solvent- and precursor-based analyses included 21 and 20 samples, respectively. Within these subsets, experimentally reported pore volumes showed substantial within-group variation and no clear trend based on solvent or precursor alone (Figure 6a). EXIT produced sample-dependent predictions from experimental XRD, although the predicted values occupied a narrower range than the measured pore volumes (Figure 6b). Given the small sample numbers, these observations are suggestive rather than conclusive. Nevertheless, they indicate that categorical synthesis labels alone do not fully account for the observed variation and that experimental XRD may provide complementary sample-specific information.

**Figure 6:**
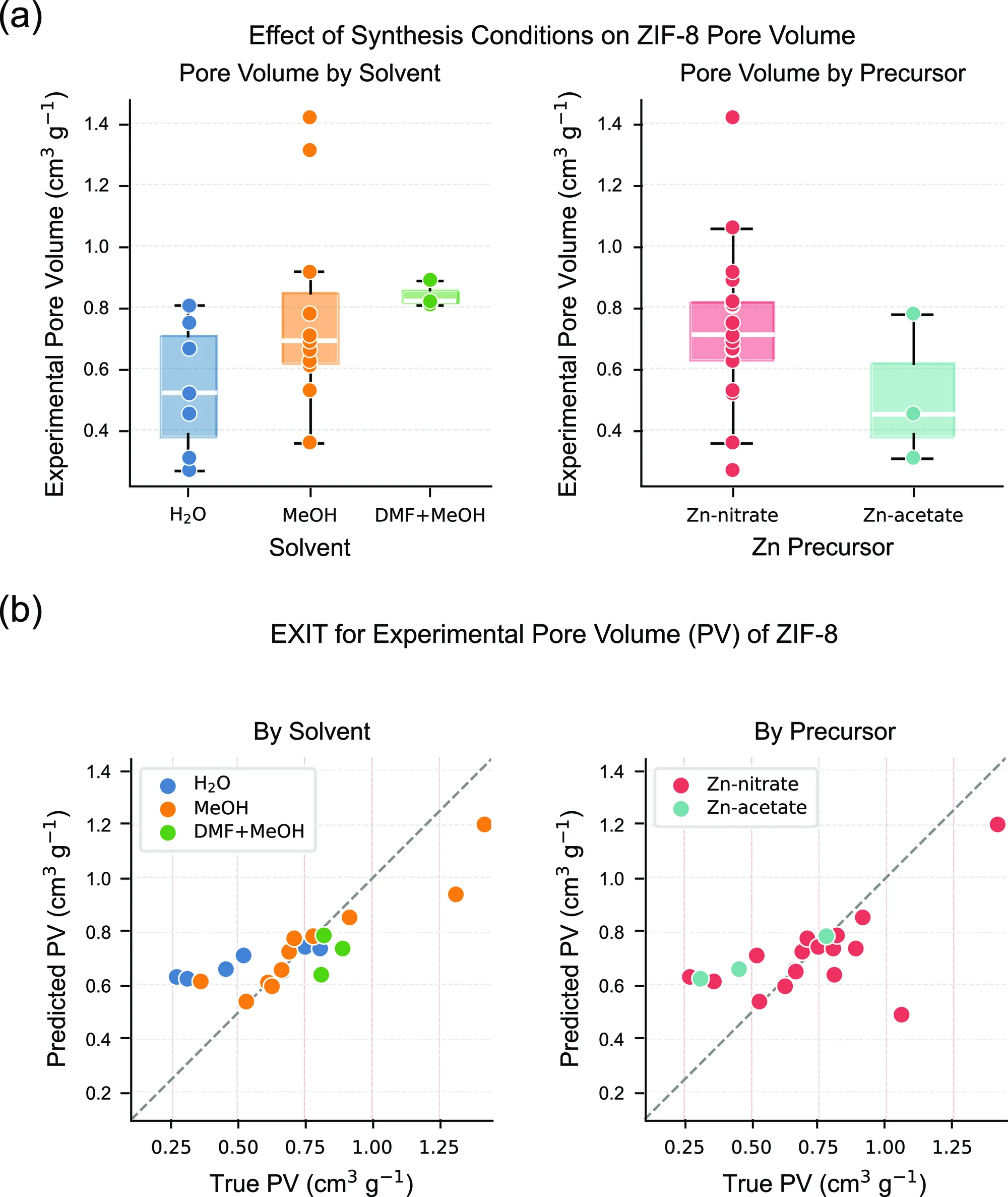
ZIF-8 samples with available synthesis condition annotations for analysis of the effects of precursor and solvent on pore volume (PV). (a) Box plots show experimentally reported PV values grouped by solvent (left, *n* = 21) and Zn precursor (right, *n* = 20). (b) Scatter plots compare predicted and true PV values for the corresponding ZIF-8 samples, grouped by solvent (left) and Zn precursor (right).

We next examined representative cases in which experimental XRD provided either strong or limited sample-specific predictive information. For MOF-5, analysis suggested that the full width at half-maximum (fwhm) of the dominant diffraction peak was associated with surface area. Smaller fwhm was consistent with larger coherent diffraction domain sizes and, in this data set, with lower surface area (Figure 7a), although in MOFs peak width can also be affected by defects, grain boundaries, and other sources of structural disorder.^38^ One possible explanation is that differences in crystalline domain structure are accompanied by differences in activation behavior, such as the efficiency of solvent removal or the degree of pore blocking, which in turn affect the accessible surface area.^39,40^ Although these differences are difficult to distinguish by visual inspection of the XRD patterns alone, EXIT appears to have learned these subtle variations. While a larger Scherrer domain size does not necessarily imply a larger crystal size of the MOF itself, the result suggests that crystallinity-related features may be linked to accessible porosity.^39^

**Figure 7:**
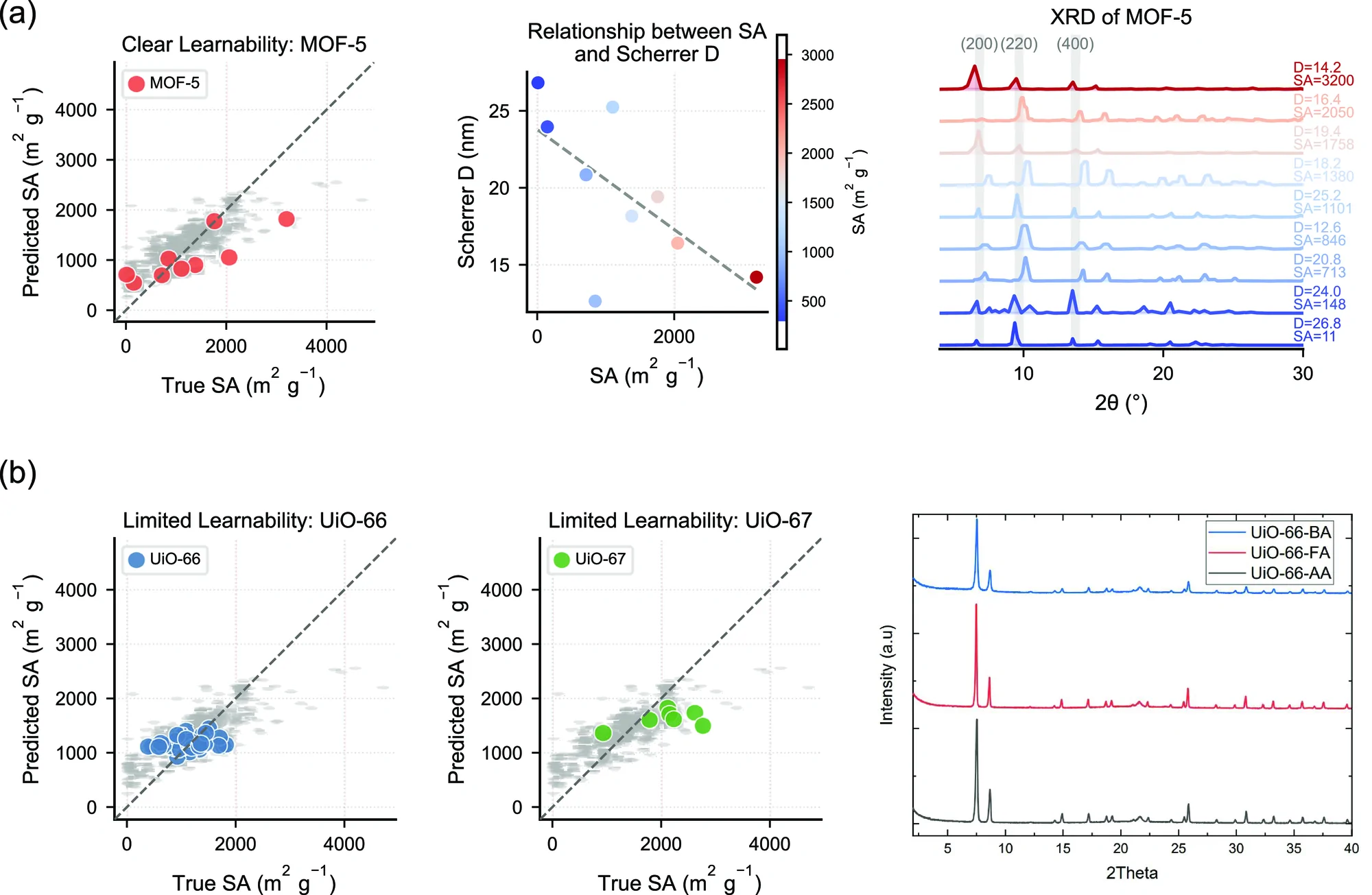
Case studies illustrating MOF-dependent behavior in experimental surface area (SA) prediction with EXIT. (a) MOF-5: predicted versus true SA values (left), relationship between Scherrer domain size and SA (middle), and experimental XRD patterns for samples with different SA values (right). (b) UiO-66 and UiO-67: predicted versus true SA values for UiO-66 (left) and UiO-67 (middle), and experimental XRD patterns of UiO-66 samples with different defect levels (right). The UiO-66 XRD patterns were adapted from ref 41. Available under a CC-BY 4.0 license. Copyright 2023 Dag Kristian Sannes, Sigurd Øien-Ødegaard, Erlend Aunan, Ainara Nova, Unni Olsbye.

In contrast, the UiO series provided representative cases in which experimental XRD provided limited additional predictive information (Figure 7b). In UiO-66 and UiO-67, missing-linker defects can alter properties such as surface area and pore volume. However, previous work has suggested that defect levels in UiO-66/67 are difficult to distinguish by XRD alone and instead require complementary analyses such as TGA, NMR, and EDX.^41^ Consistent with this, the XRD patterns shown in Figure 7b (right) exhibit only minor differences despite differences in defect levels. In such cases, where the experimentally relevant variation is not clearly reflected in XRD, the predictive benefit of incorporating experimental XRD is expected to be limited.

Taken together, these analyses show that experimental XRD can provide sample-relevant structural information beyond idealized framework representations alone, although its usefulness depends on whether the experimentally important variation is reflected in the diffraction pattern. Finally, although EXIT requires experimental input in the form of measured XRD, this dependence remains practically attractive because powder XRD is a routine and comparatively accessible characterization method in MOF research. In many workflows, diffraction data can be acquired earlier and more readily than detailed adsorption measurements, allowing the model to use a widely available structural signal to inform sample-aware property prediction. Rather than replacing experiment, this framework suggests a way to use early stage characterization to prioritize samples for more resource-intensive follow-up measurements.

## Discussion

In this work, we introduced EXIT, a multimodal Transformer framework that integrates MOFid and XRD to enable experiment-aware prediction of MOF properties across hypothetical and experimental settings. Pretraining on one million hypothetical MOFs showed that the model can learn transferable multimodal representations from chemical identity and diffraction-derived structural information. On simulated-XRD benchmarks, the pretrained model achieved improved downstream performance for thermal decomposition temperature and CH_4_ uptake prediction compared with existing approaches and with the model trained from scratch. Applying EXIT to literature-derived experimental data sets further demonstrated that incorporating experimental XRD improves prediction of surface area and pore volume compared with models without experimental XRD. Attention analyses and case studies further showed that EXIT can assign different predictions to samples of the same MOF when experimental XRD is provided and that the predictive benefit of XRD depends on whether the relevant variation is reflected in the diffraction patterns. The present study should be viewed as a proof-of-concept rather than a definitive benchmark. The limited data set size and literature-derived nature of the property records introduce variability that cannot be fully controlled, including differences in activation procedures, sample handling, measurement conditions, and data-analysis protocols across studies. Consequently, some residual prediction error may reflect uncontrolled experimental variability as well as limitations of the model. Future work should therefore prioritize larger paired data sets containing machine-readable XRD patterns, standardized experimental metadata, consistently measured property values, and replicate measurements, thereby enabling sample-aware prediction to be extended to a broader range of experimental properties. Taken together, these findings establish EXIT as a practical step from framework-level prediction toward sample-level prediction in porous materials informatics and highlight the value of incorporating experimental characterization into data-driven materials discovery.

## Methods

### Multimodal Transformer

EXIT adopts a multimodal architecture that takes MOFid sequences and XRD patterns as inputs. The MOFid vocabulary contains 3,621 unique tokens, and each sequence includes up to 512 tokens. MOFid sequences longer than 512 tokens were truncated, whereas shorter sequences were padded to 512 tokens and masked in self-attention. The XRD pattern is represented by normalized intensities sampled from 2θ = 0–50° at a 0.01° interval, resulting in a 5,000-dimensional input vector. The XRD signal is divided into nonoverlapping 1D patches of size 20, and each patch is projected to a 768-dimensional embedding using a linear patch embedding layer. MOFid tokens use fixed sinusoidal positional encodings, whereas XRD patch embeddings use learnable positional embeddings. The resulting MOFid and XRD token embeddings are augmented with modality-specific token-type embeddings and concatenated along the sequence dimension to form a single multimodal token sequence, which is then passed through the Transformer blocks. These multimodal Transformer blocks have a hidden dimension of 768 and consist of 12 layers with 12 attention heads per layer. Each attention head has a dimension of 64. Each block uses residual connections and layer normalization around the self-attention and feed-forward layers. For void-fraction prediction, the final hidden representation of the [CLS] token is passed through a pooling layer and a linear regression head.

### Pretraining

To pretrain EXIT, we constructed a large-scale data set by combining hypothetical MOFs generated with PORMAKE,^11^ a Python library that builds porous materials from predefined topologies and building blocks, and MOF structures collected from the hMOF, CoRE MOF, and QMOF databases. MOFid representations were extracted from these structures, and their corresponding XRD patterns were simulated using the pymatgen library. For the pretraining tasks, we employed masked language modeling (MLM) on the MOFid sequences and the prediction of the void fraction, which was computed using Zeo++.^42^ In the MLM task, 15% of MOFid tokens were randomly selected for prediction. Following the standard BERT-style masking procedure, 80% of the selected tokens were replaced with [MASK], 10% were replaced with random tokens, and 10% were left unchanged. We used the AdamW optimizer with a learning rate of 10^–4^ and a weight decay of 10^–2^, and trained the model for 500 epochs with a batch size of 256. The data set was randomly split into training and test sets at a ratio of 0.95:0.05. A polynomial decay scheduler with a warm-up ratio of 0.05 was applied during training.

### Fine-Tuning

The pretrained EXIT model was fine-tuned under two downstream settings: T_D_ and CH_4_ uptake prediction using simulated XRD, and surface area and pore volume prediction using literature-derived experimental XRD. Experimental XRD was used only for the latter fine-tuning tasks and was not included during pretraining.

For downstream prediction of thermal decomposition temperature (T_D_) and CH_4_ uptake using simulated XRD, we fine-tuned the model using the AdamW optimizer with a learning rate of 1 × 10^–4^ and a weight decay of 10^–2^. The data set was randomly split into training, validation, and test sets at a ratio of 0.65:0.15:0.20. For prediction of surface area and pore volume using experimental XRD, the learning rate was set to 5 × 10^–5^, and the data set was split into training, validation, and test sets at a ratio of 0.8:0.1:0.1. In all cases, the model was trained for 30 epochs with a batch size of 128. A polynomial decay scheduler with a warm-up ratio of 0.05 was applied during training. All fine-tuning experiments were performed using a fixed random seed of 0. For experimental surface area and pore volume prediction, we additionally performed 9-fold cross-validation by dividing the full data set into ten folds, fixing one fold as the test set and using the remaining nine folds for cross-validation.

### MatGD

XRD data were extracted using MatGD, a graph-mining extraction agent developed in our group by combining with GPT-5.4,^43^ a multimodal large language model. We first retrieved figures from papers in the L2M3 database whose DOIs were associated with reported surface area or pore volume values. MatGD then used figure captions to filter for figures containing XRD patterns. For each selected figure, the model was used to identify the correspondence between individual data lines and their associated MOF entities. The extracted XRD traces were subsequently digitized, producing experimental XRD-property pairs that connect digitized XRD patterns with MOF property values curated in the L2M3 database.

To standardize the extracted signals, each digitized trace was processed through centerline extraction, baseline correction, normalization, and resampling. Because graph extraction can return a band of pixel coordinates rather than a single curve, the extracted points were first binned along the 2θ axis and reduced to a one-dimensional centerline using the median intensity in each bin. In regions containing sharp peaks, the upper part of the extracted pixel band was used instead of the median to avoid artificially flattening Bragg reflections, and the resulting centerline was smoothed with a Savitzky–Golay filter. Baseline correction was then evaluated using three asymmetric least-squares-based methods implemented in the ramanspy library, namely arPLS, AsLS, and airPLS, with default parameters.^44^ For each trace, the optimal baseline was selected using a composite score calculated as a weighted sum of three terms: the average negative-intensity penalty over the entire corrected trace, the mean absolute deviation between the estimated baseline and its rolling lower-percentile envelope, and the mean absolute second derivative of the baseline. These terms penalize oversubtraction, baselines that rise into peak regions, and excessive baseline roughness, respectively. The selected baseline was subtracted, and the corrected trace was min–max normalized to the range of 0–1. The normalized trace was then resampled by linear interpolation onto a uniform grid from 2θ = 0–50° at 0.01° intervals, yielding a 5,000-dimensional XRD input vector. All extracted XRD–property pairs were further manually inspected, and samples containing errors or ambiguities were removed during the curation process. All digitized PXRD patterns were manually compared with their corresponding source figures for validation.

## Supplementary Material



## Data Availability

The code is available at https://github.com/seunghhs/EXIT.git, and the corresponding data set is available at 10.5281/zenodo.19675999

## Notes

Distribution of experimentally mined MOF property data; prediction results of the best-performing EXIT model for experimental surface area and pore volume, grouped by MOF; performance of the pretraining tasks on the hypothetical MOF data set; test-set performance on downstream fine-tuning tasks with different pretraining tasks; and cross-validation results for experimental surface area and pore volume prediction (PDF)
