# Supplementary material for: Multimodal Transformer for Sample-Aware Prediction of Metal–Organic Framework Properties
Source: ACS Cent Sci. 2026 Sep 1;12(9):1504–14. doi: 10.1021/acscentsci.6c01049 (PMC13614057; doi:10.1021/acscentsci.6c01049)
Supplement: Supplementary file 1 [file oc6c01049_si_001.pdf]

Supporting Information

Multimodal Transformer for Sample-Aware Prediction of Metal-Organic Framework Properties

Seunghee Han[1‡], Jaewoong Lee[2‡], and Jihan Kim[1]*

1 Department of Chemical and Biomolecular Engineering, Korea Advanced Institute of Science and Technology, Daejeon 34141, Republic of Korea.

2 Department of Materials Science and Engineering, Korea Advanced Institute of Science and Technology, Daejeon 34141, Republic of Korea.

*Corresponding author: jihankim@kaist.ac.kr

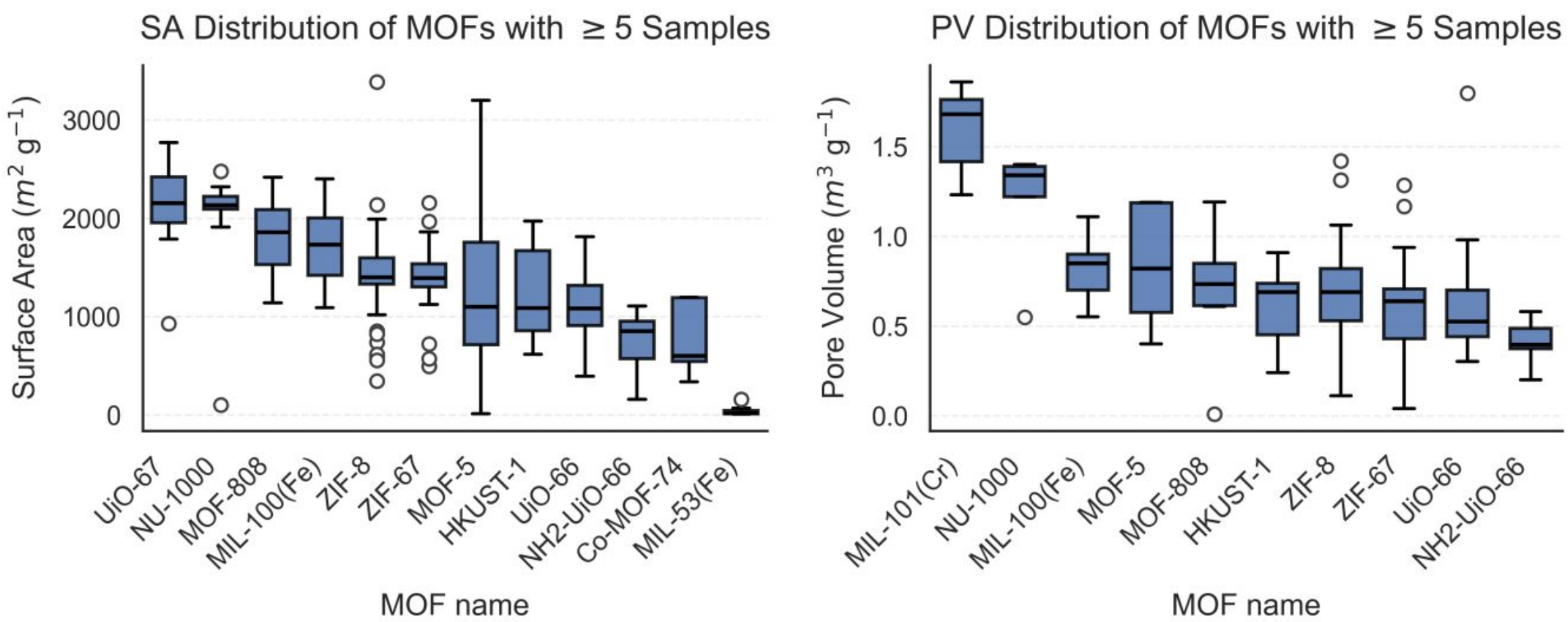

Figure S1 Distribution of experimentally mined MOF property data. Box plots show surface area (SA) and pore volume (PV) for MOFs with at least five samples among those with paired XRD patterns, CIF files for MOFid generation, and reported property values. The dataset comprises 311 SA samples across 84 MOFs and 181 PV samples across 49 MOFs. The spread within each MOF reflects variability in experimentally reported properties for the same nominal MOF identity.

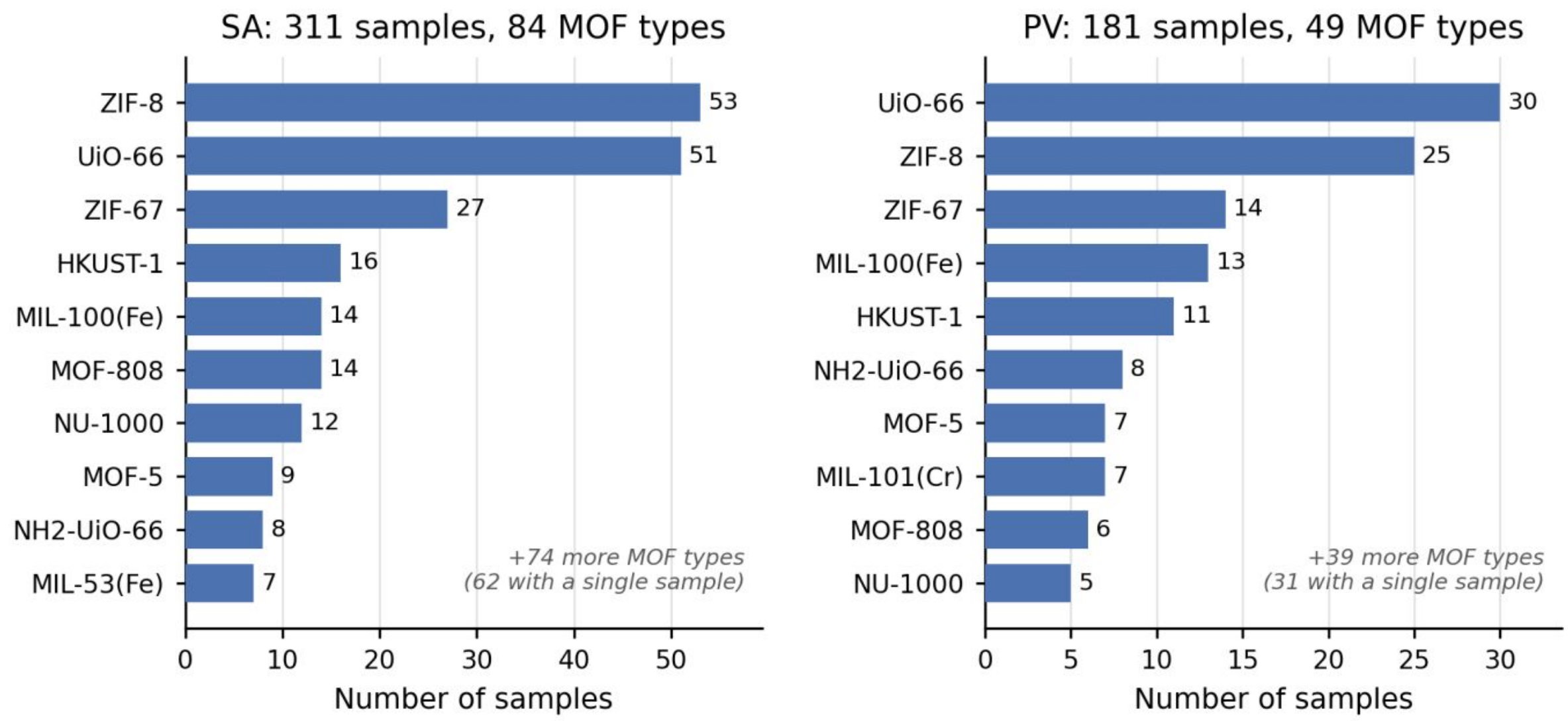

Figure S2 Distribution of sample counts per MOF type in the fine-tuning datasets for surface area (SA) (left) and pore volume (PV) (right). Bars show the ten most frequently occurring MOF types, ranked by the number of experimental samples. Both datasets are dominated by a few high-frequency frameworks, such as ZIF-8, UiO-66, and ZIF-67, followed by a long tail of low-frequency frameworks: beyond the ten shown, 74 additional MOF types (62 represented by a single sample) for SA and 39 additional types (31 single-sample) for PV.

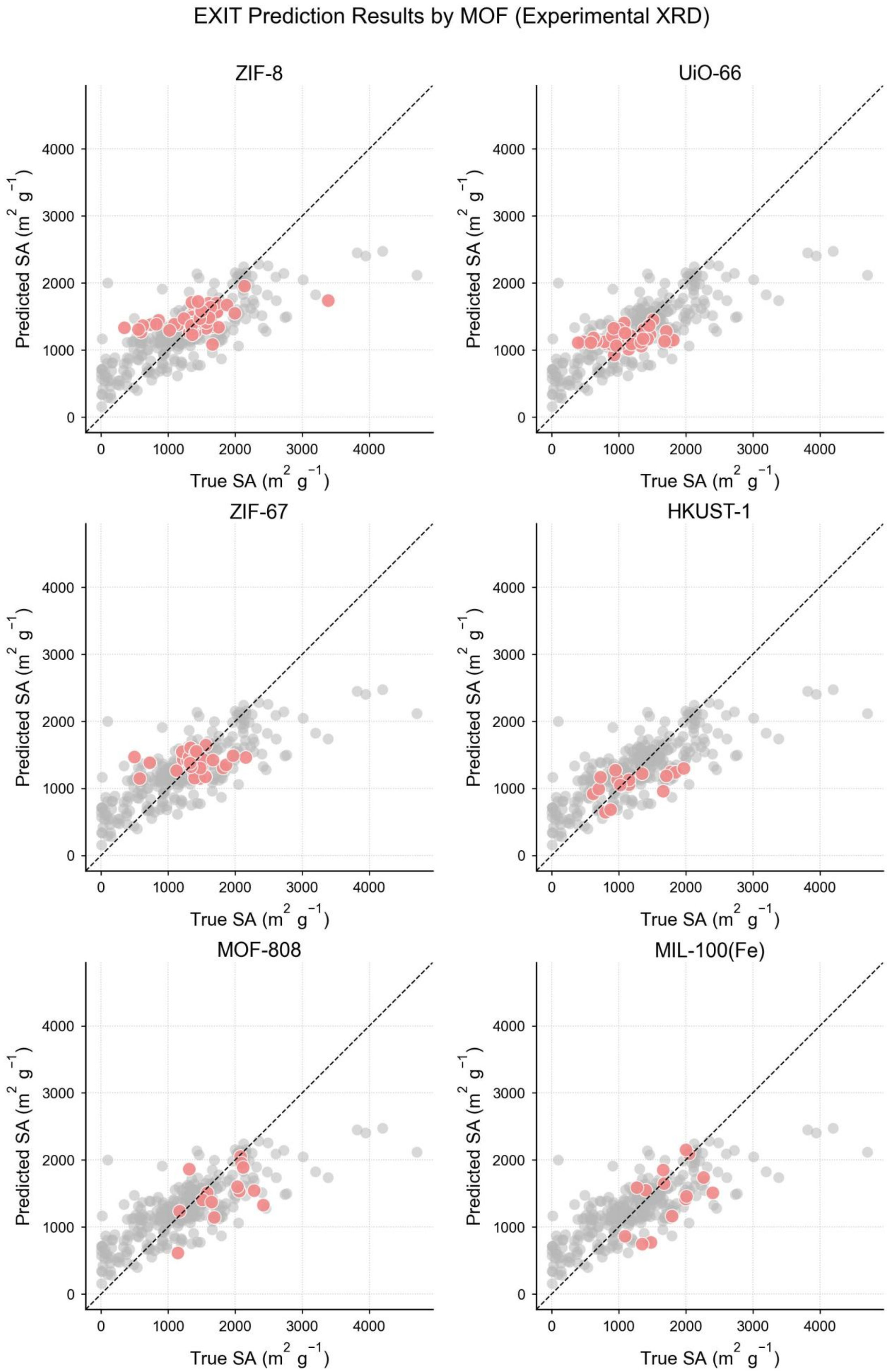
EXIT Prediction Results by MOF (Experimental XRD)
ZIF-8
UiO-66
ZIF-67
HKUST-1
MOF-808
MIL-100(Fe)
Predicted SA (m^2 g^{-1})
True SA (m^2 g^{-1})
0
1000
2000
3000
4000

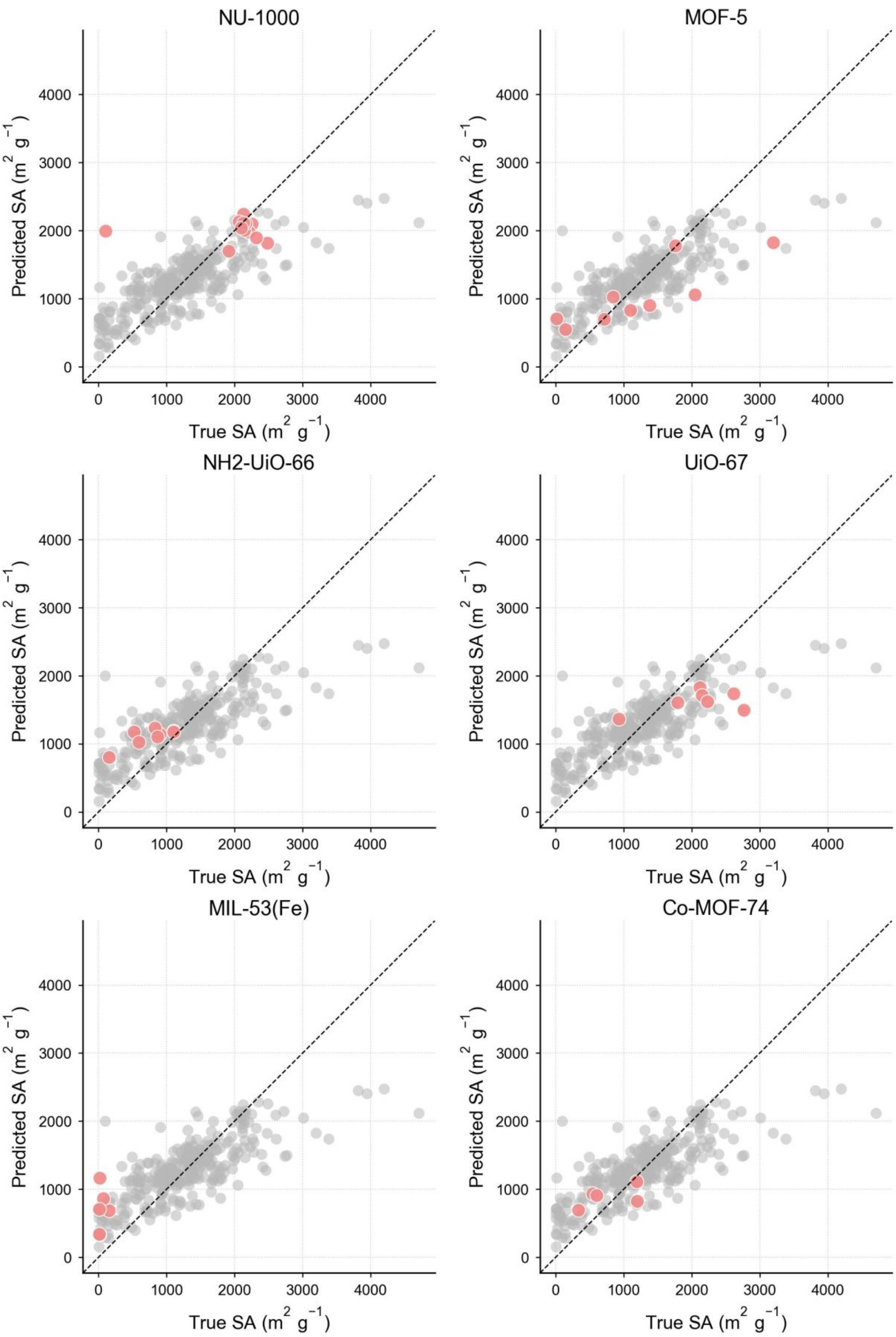

Figure S3 Prediction results of the best-performing EXIT model for experimental surface area (SA), grouped by MOF. Scatter plots show predicted versus true SA values using the combined training, validation, and test data. Only MOFs with at least five samples are included. Red points indicate samples of the highlighted MOF, while gray points represent all other samples. The dashed line represents the identity line.

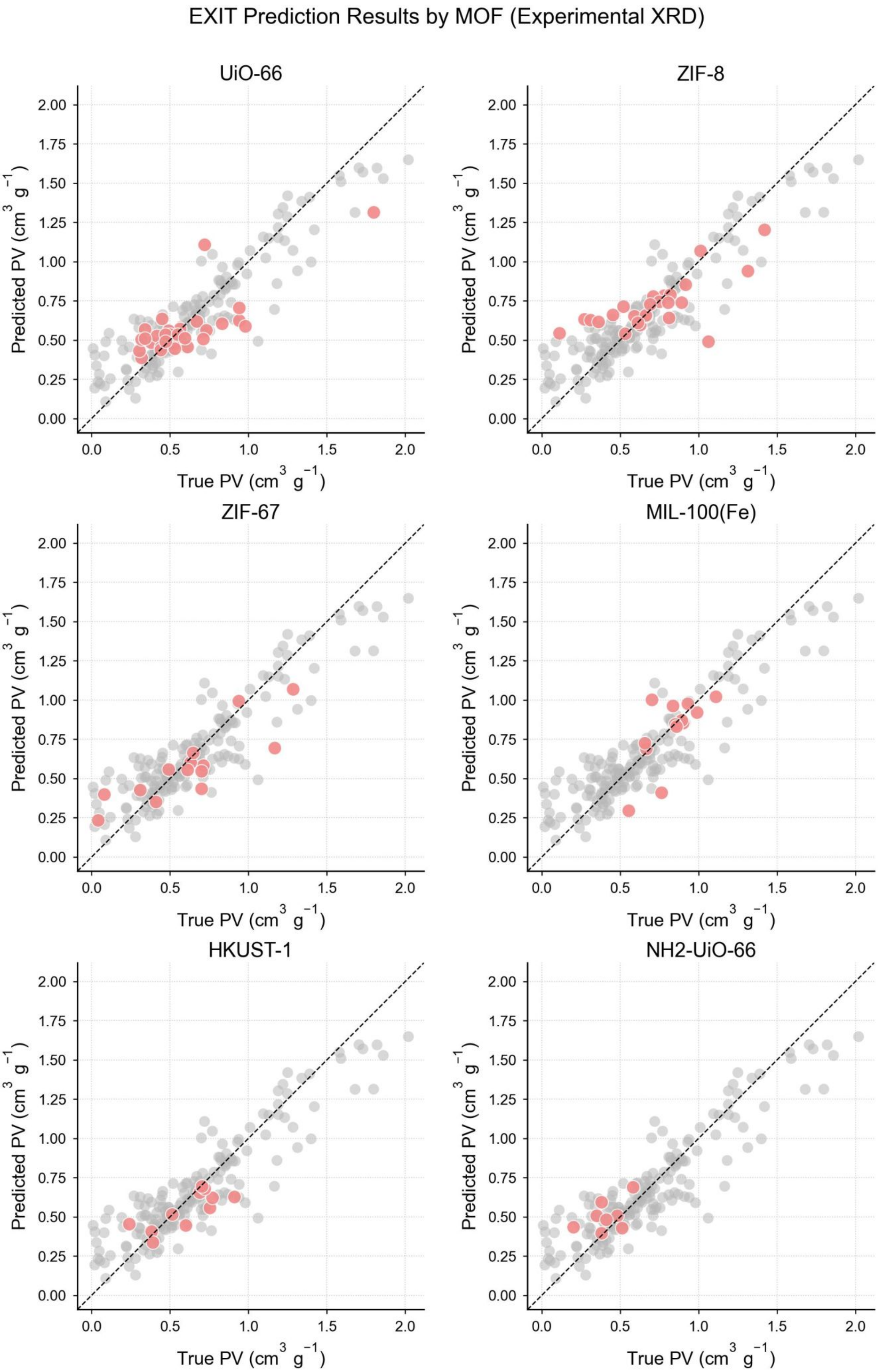
EXIT Prediction Results by MOF (Experimental XRD)
UiO-66
ZIF-8
ZIF-67
MIL-100(Fe)
HKUST-1
NH2-UiO-66
Predicted PV (cm3 g−1)
True PV (cm3 g−1)
0.00
0.25
0.50
0.75
1.00
1.25
1.50
1.75
2.00
0.0
0.5
1.0
1.5
2.0

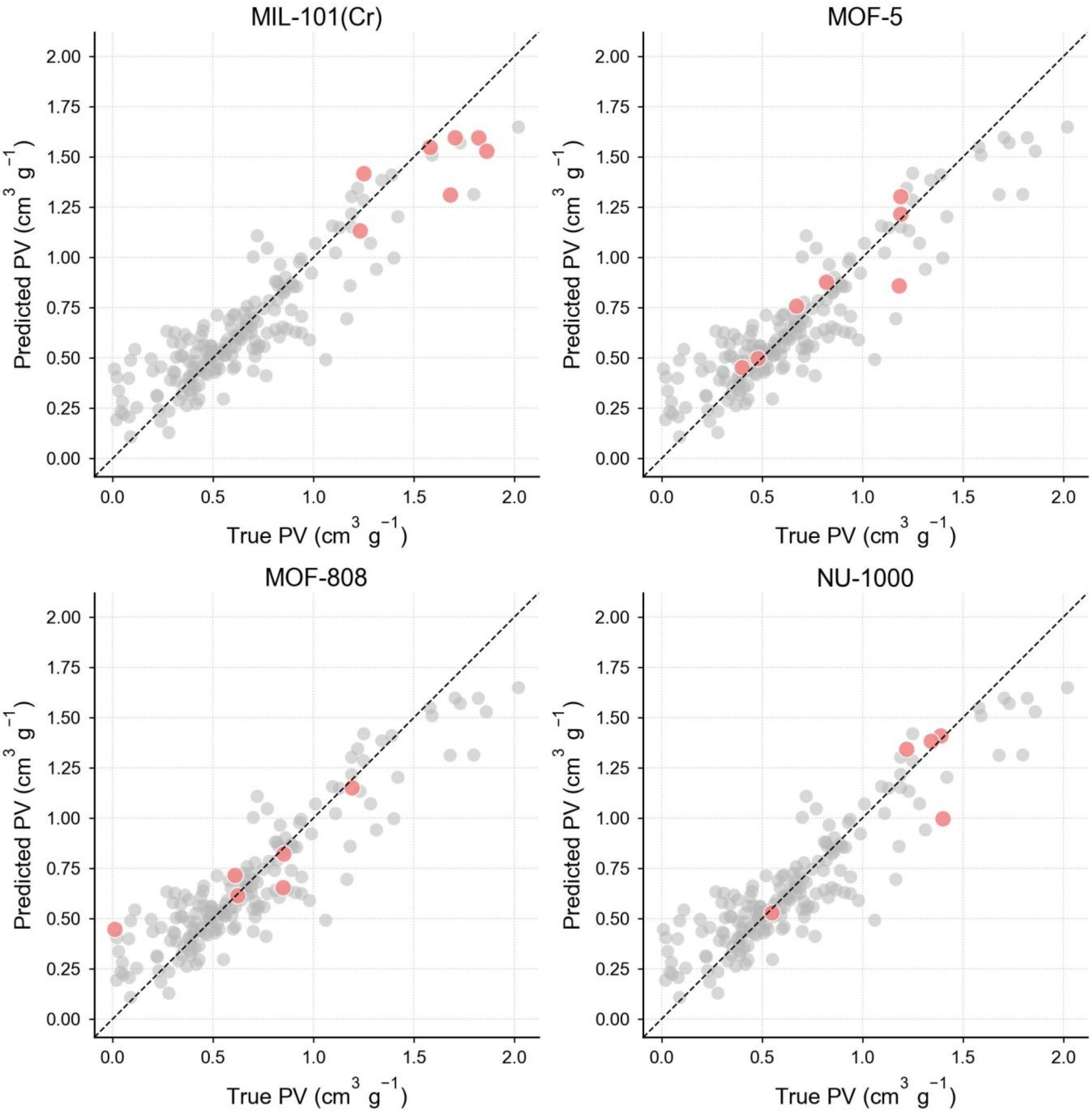

Figure S4 Prediction results of the best-performing EXIT model for experimental pore volume (PV), grouped by MOF. Scatter plots show predicted versus true PV values using the combined training, validation, and test data. Only MOFs with at least five samples are included. Red points indicate samples of the highlighted MOF, while gray points represent all other samples. The dashed line represents the identity line.

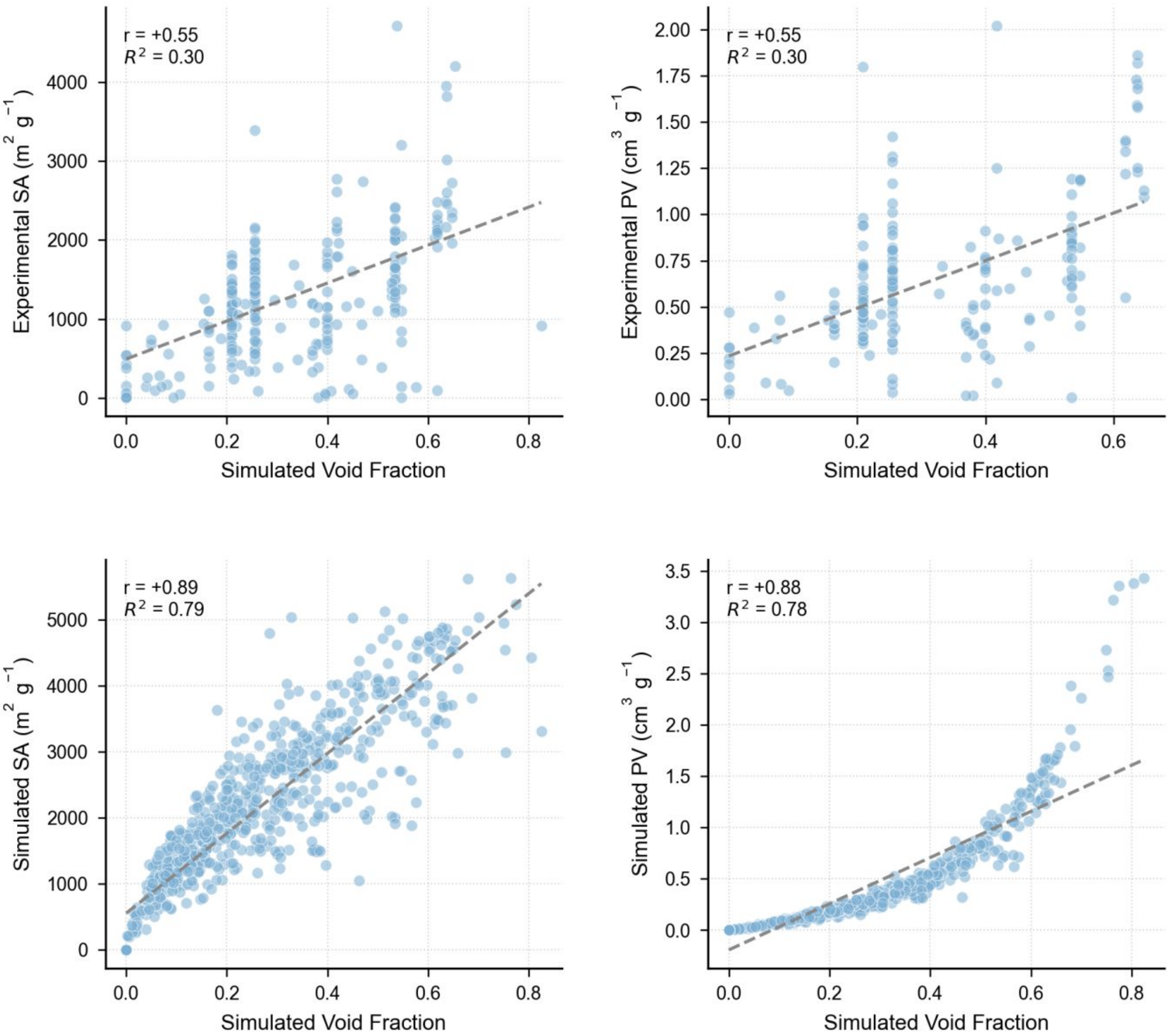

Figure S5 Relationship between framework-derived accessible void fraction and experimental and simulated surface area and pore volume. The top panels compare accessible void fraction (AVF), computed from idealized MOF framework structures using Zeo++, with the experimentally measured surface area (left) and pore volume (right) of samples used for EXIT fine-tuning. The bottom panels compare AVF with the corresponding framework-derived simulated surface area (left) and pore volume (right). The dashed lines indicate ordinary least-squares linear fits, with Pearson's $\boldsymbol{r}$ and $\boldsymbol{R^2}$ reported in each panel. The variation in experimental properties at a given AVF reflects sample-to-sample variability that is not captured by this framework-level descriptor.

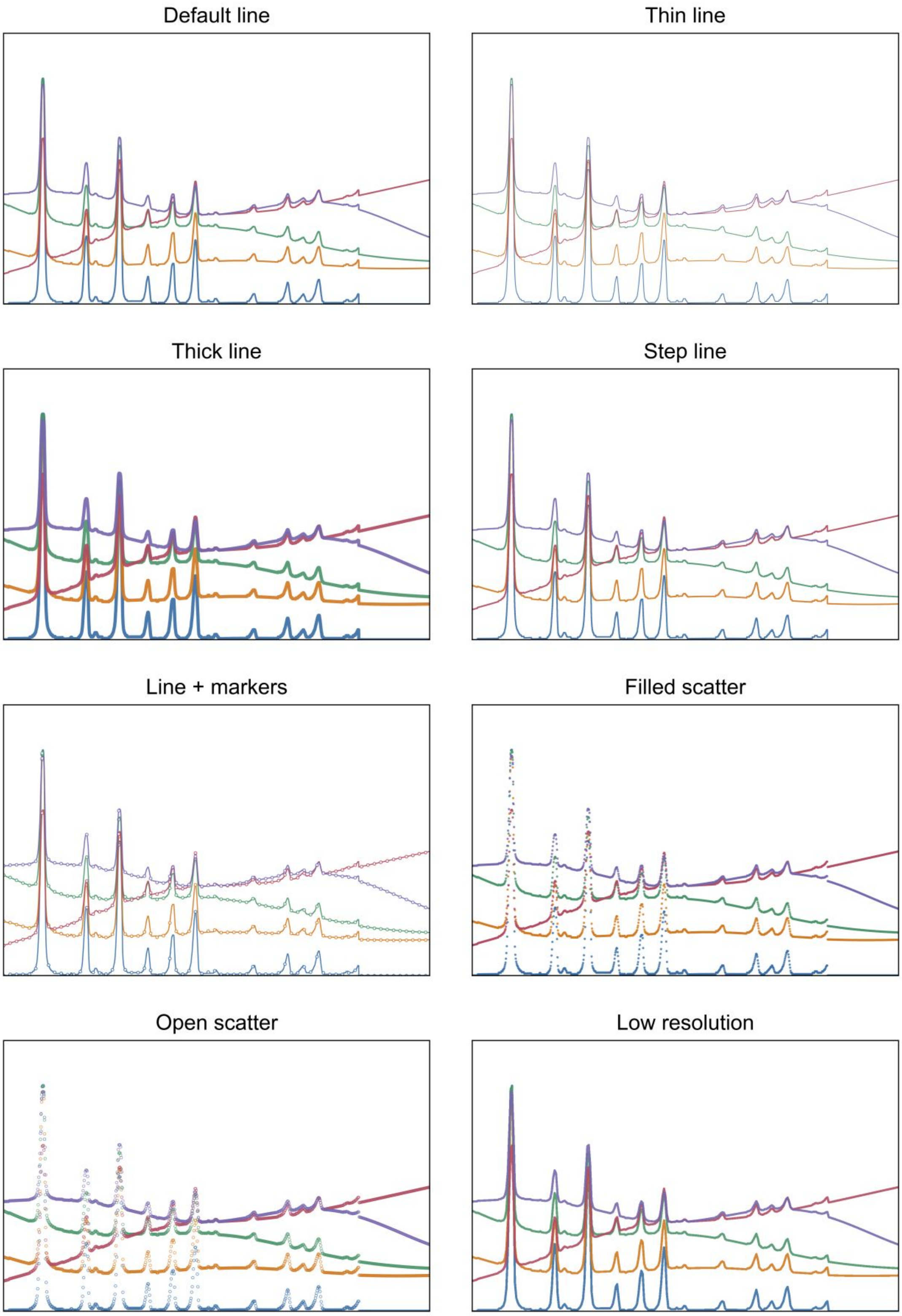

Figure S6 Synthetic PXRD pattern variations used to evaluate the robustness of MatGD extraction and preprocessing code. A single PXRD pattern was augmented by rendering it under five baseline conditions and eight plotting styles

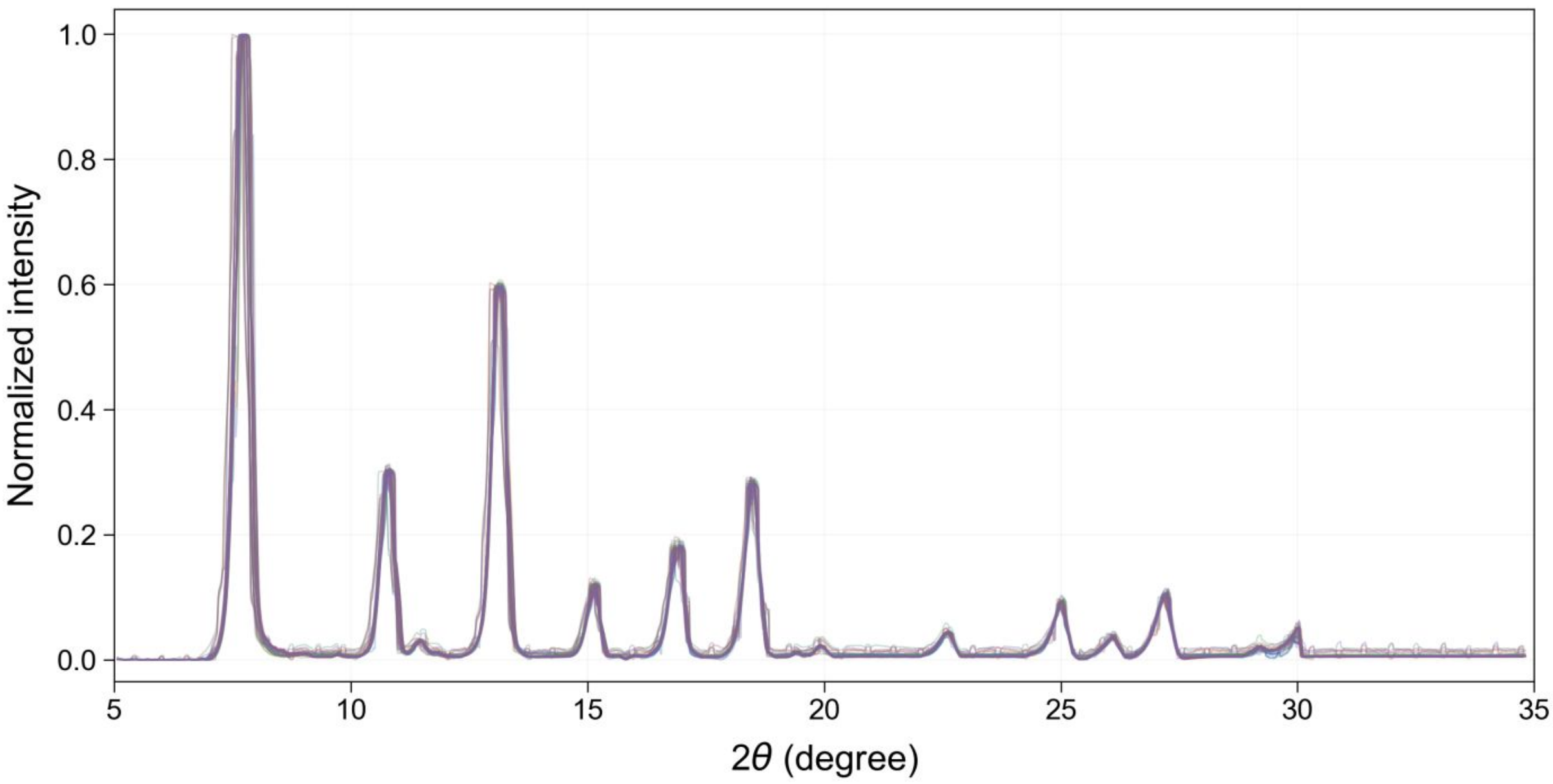

Figure S7 Overlay of the augmented PXRD patterns after MatGD extraction and processing code.

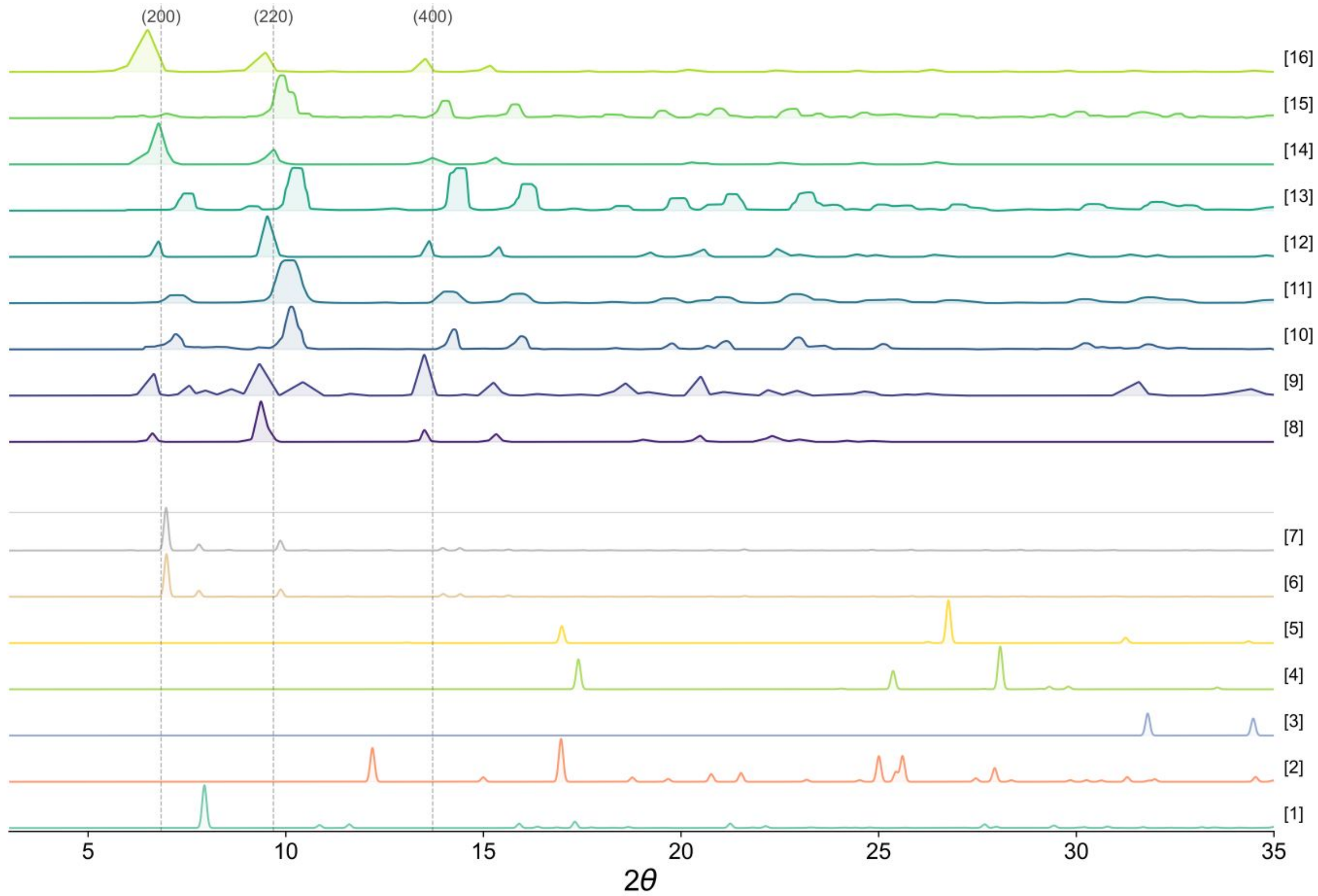

Figure S8 Simulated PXRD patterns of MOF-5 related candidate phases and experimental MOF-5 PXRD traces extracted from the dataset.

Trace indices are listed from bottom to top:
[1] $Zn_3OH_2BDC_2 \cdot$ 2DEF CCDC 267120
[2] ZnBDC $\cdot H_2O_2$ COD 7115213
[3] ZnO wurtzite COD 9004180
[4] H_2BDC terephthalic-acid triclinic CCDC 1269130
[5] H_2BDC terephthalic-acid monoclinic CCDC 15487
[6] CMOF-5 chiral P21, doi:10.1021/jacs.5b11150
[7] CMOF-5 chiral/solvated P213, doi:10.1021/jacs.5b11150
[8] Experimental MOF-5, doi:10.1016/j.dyepig.2021.109545
[9] Experimental MOF-5, doi:10.1016/j.ijbiomac.2019.12.007
[10] Experimental MOF-5, doi:10.1016/j.arabjc.2017.01.012
[11] Experimental MOF-5, doi:10.1016/j.colsurfa.2018.08.009
[12] Experimental MOF-5, doi:10.1016/j.jallcom.2018.10.383
[13] Experimental MOF-5, doi:10.1016/j.cej.2023.141663
[14] Experimental MOF-5, doi:10.1016/j.ijhydene.2010.04.066
[15] Experimental MOF-5, doi:10.1016/j.carbon.2017.07.056
[16] Experimental MOF-5, doi:10.1021/cm502399q

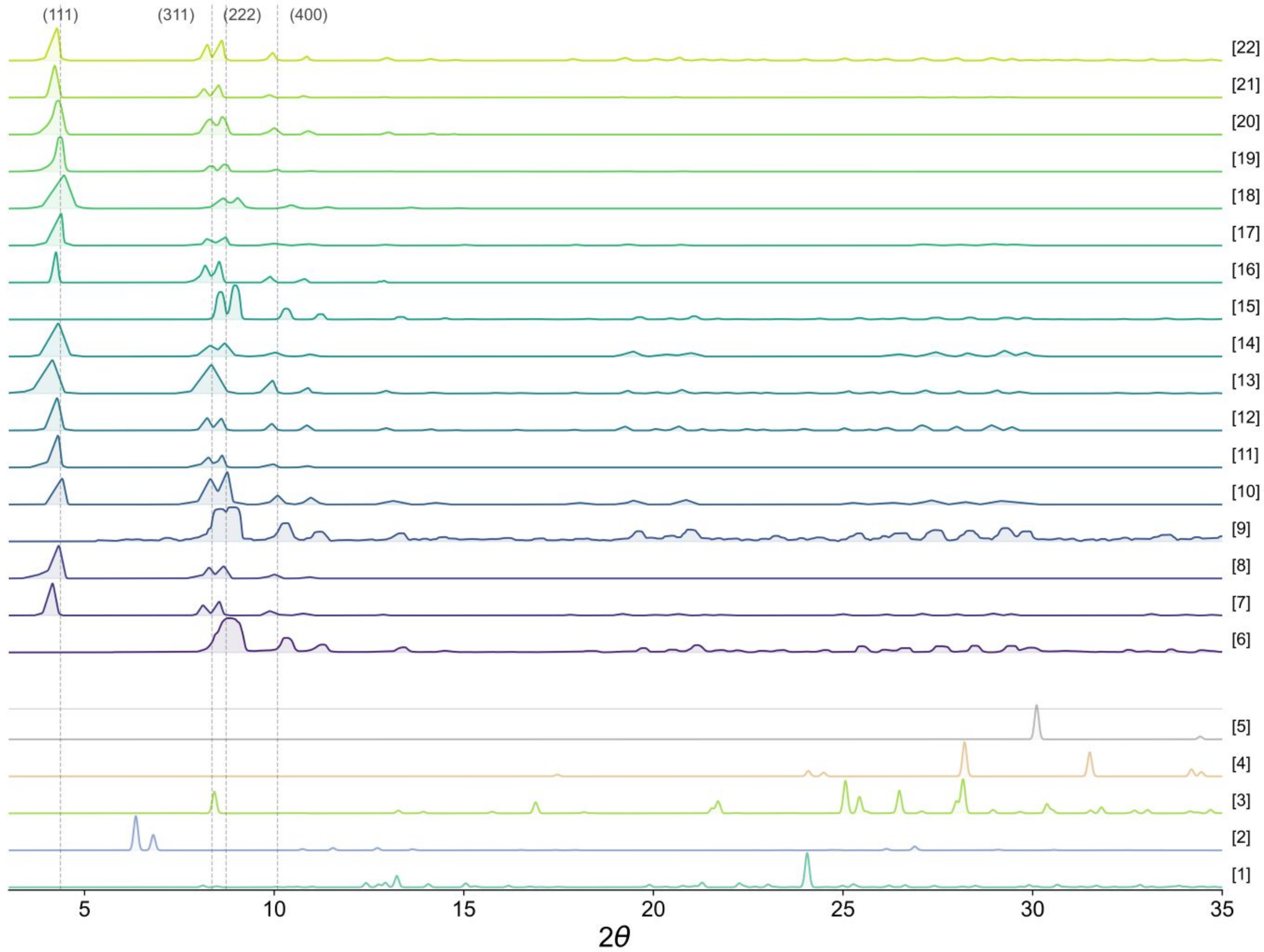

Figure S9 Simulated PXRD patterns of MOF-808 related candidate phases and experimental MOF-808 PXRD traces extracted from the dataset.

Trace indices are listed from bottom to top:
[1] H_3BTC / trimesic acid COD 4111695, [2] H_3BTC polymorph COD 1555266
[3] H_3BTC dihydrate COD 2206049, [4] ZrO_2 monoclinic COD 1528984
[5] ZrO_2 tetragonal COD 1526427
[6] Experimental MOF-808, doi:10.1016/j.cej.2022.134830
[7] Experimental MOF-808, doi:10.1021/acs.inorgchem.1c00185
[8] Experimental MOF-808, doi:10.1016/j.cej.2022.135650
[9] Experimental MOF-808, doi:10.1021/acs.inorgchem.8b01097
[10] Experimental MOF-808, doi:10.1016/j.cej.2023.142480
[11] Experimental MOF-808, doi:10.1016/j.cej.2019.01.044
[12] Experimental MOF-808, doi:10.1021/jacs.1c03283
[13] Experimental MOF-808, doi:10.1016/j.gce.2022.01.007
[14] Experimental MOF-808, doi:10.1021/acsami.0c14946
[15] Experimental MOF-808, doi:10.1021/acs.inorgchem.2c01444
[16] Experimental MOF-808, doi:10.1021/acsaem.2c01101
[17] Experimental MOF-808, doi:10.1021/acsami.0c21007
[18] Experimental MOF-808, doi:10.1021/acs.chemmater.2c03426
[19] Experimental MOF-808, doi:10.1021/acs.chemmater.1c02174
[20] Experimental MOF-808, doi:10.1021/acsaem.0c00399
[21] Experimental MOF-808, doi:10.1021/acscatal.3c00398
[22] Experimental MOF-808, doi:10.1021/acsami.1c23458

Table S1 Performance of the pre-training tasks after pre-training on the 1 million hypothetical MOF dataset, evaluated on the test set. MOFid prediction is reported as accuracy, and void fraction prediction is reported as R^2 score.

Pre-training Tasks	Test Performance
MOFid (Accuracy)	0.36
Void Fraction (R^2 Score)	0.98

Table S2 Test-set performance on downstream fine-tuning tasks using simulated XRD, evaluated to assess the effect of different pre-training targets. Models were pre-trained with MOFid prediction, void fraction prediction, or both tasks jointly. Performance is reported as mean absolute error (MAE) for thermal decomposition temperature (T_D) and CH_4 adsorption at 298 K and 2.5 bar, where lower values indicate better performance.

	Thermal Decomposition Temperature (T_D) (MAE)	CH_4 Adsorption (298K, 2.5 bar) (MAE)
MOFid	44.67	0.20
Void Fraction	50.86	0.19
MOFid + Void Fraction	**44.58 (↓)**	**0.17 (↓)**

Table S3 9-fold cross-validation results for experimental surface area (SA) prediction. The full dataset was divided into ten folds, with one fixed as the test set and the remaining nine used for cross-validation. Performance is reported as R^2, mean absolute error (MAE), and Pearson correlation coefficient. The model incorporating experimental XRD achieved better average performance than the model without experimental XRD across all metrics.

	With Experimental XRD	Without Experimental XRD
R^2 score (↑)	$\mathbf{0.45 \pm 0.11}$	0.20 ± 0.10
MAE (↓)	$\mathbf{360 \pm 35}$	420 ± 15
Pearson Coefficient (↑)	$\mathbf{0.71 \pm 0.06}$	0.56 ± 0.04

Table S4 9-fold cross-validation results for experimental pore volume (PV) prediction. The full dataset was divided into ten folds, with one fixed as the test set and the remaining nine used for cross-validation. Performance is reported as R^2, mean absolute error (MAE), and Pearson correlation coefficient. The model incorporating experimental XRD achieved better average performance than the model without experimental XRD across all metrics.

	With Experimental XRD	Without Experimental XRD
R^2 score (↑)	$\mathbf{0.47 \pm 0.08}$	0.12 ± 0.10
MAE (↓)	$\mathbf{0.23 \pm 0.01}$	0.28 ± 0.02
Pearson Coefficient (↑)	$\mathbf{0.75 \pm 0.04}$	0.60 ± 0.03

Table S5 Performance of EXIT after MLM-only pre-training. Values are reported as mean ± standard deviation across the nine fine-tuning folds for models using sample-specific experimental XRD or framework-derived simulated XRD. Bold values indicate the better-performing result for each metric.

Surface Area (SA)	With Experimental XRD	Without Experimental XRD
R^2 score (↑)	0.28 ± 0.18	$\mathbf{0.29 \pm 0.13}$
MAE (↓)	$\mathbf{386 \pm 54}$	388 ± 27
Pearson Coefficient (↑)	0.61 ± 0.14	$\mathbf{0.62 \pm 0.06}$

Pore Volume (PV)	With Experimental XRD	Without Experimental XRD
R^2 score (↑)	$\mathbf{0.12 \pm 0.08}$	0.03 ± 0.13
MAE (↓)	0.30 ± 0.02	$\mathbf{0.29 \pm 0.02}$
Pearson Coefficient (↑)	$\mathbf{0.60 \pm 0.06}$	0.53 ± 0.09

Table S6 Effect of train–test literature-source DOI overlap on surface-area (SA) and pore-volume (PV) prediction. Performance was reevaluated after removing from the original fixed held-out test set all samples whose source DOI was also represented in the corresponding training partition. Results are reported as mean ± standard deviation across the nine fine-tuning folds for models using experimental or simulated XRD. Values in parentheses indicate changes relative to performance on the original test set. The minimal changes indicate that the limited literature-source overlap did not materially affect the reported performance.

Test Dataset	DOI/source
Surface Area	$2.9 \pm 0.33 / 39$
Pore Volume	$1.8 \pm 0.44 / 21$

Surface Area (Clean, DOI-disjoint)	With Experimental XRD	Without Experimental XRD
R^2 score (↑)	$\mathbf{0.46} \pm \mathbf{0.09}$ $(+\mathbf{0.01})$	0.24 ± 0.08 $(+0.04)$
MAE (↓)	$\mathbf{357} \pm \mathbf{30}$ $(-\mathbf{3.73})$	411 ± 12 (-8.35)
Pearson Coefficient (↑)	$\mathbf{0.71} \pm \mathbf{0.06}$ $(+\mathbf{0.00})$	0.57 ± 0.03 $(+0.01)$

Pore Volume (Clean, DOI-disjoint)	With Experimental XRD	Without Experimental XRD
R^2 score (↑)	$\mathbf{0.47} \pm \mathbf{0.08}$ $(-\mathbf{0.01})$	0.11 ± 0.08 (-0.00)
MAE (↓)	$\mathbf{0.24} \pm \mathbf{0.02}$ $(+\mathbf{0.01})$	0.29 ± 0.02 $(+0.01)$
Pearson Coefficient (↑)	$\mathbf{0.75} \pm \mathbf{0.05}$ $(+\mathbf{0.01})$	0.60 ± 0.03 (-0.00)

Table S7 Distribution of solvent and Zn precursor annotations across the 25 ZIF-8 records. Bold entries indicate the categories included in **Figure 6**. Categories with unknown annotations or represented by a single sample were excluded from the grouped analyses.

Solvent	n	Precursor	n
**MeOH**	**11**	**Zn-nitrate**	**17**
**H2O**	**7**	**Zn-acetate**	**3**
**DMF+MeOH**	**3**	Zn-hydroxide	1
DMF+H2O	1	Unknown	4
Unknown	3		
Sum	25		25

Table S8 Quantitative comparison of XRD digitization accuracy between MatGD and Plot2Spec. The corresponding XRD comparison groups are shown in Figure S6.

MatGD			
x(°2θ)	y(normalized)	Mean absolute x error	Mean absolute y error
7.80	1.0000	0.0488	0.0000
10.85	0.3006	0.0478	0.0033
13.19	0.5957	0.0356	0.0029
15.16	0.1218	0.0320	0.0022
16.98	0.1793	0.0120	0.0038
18.52	0.2834	0.0188	0.0030
**Plot2Spec**			
x(°2θ)	y(normalized)	Mean absolute x error	Mean absolute y error
7.80	1.0000	0.2014	0.1300
10.85	0.3006	0.2300	0.1164
13.19	0.5957	0.2124	0.1292
15.16	0.1218	0.2273	0.1071
16.98	0.1793	0.2135	0.1142
18.52	0.2834	0.1886	0.1221
